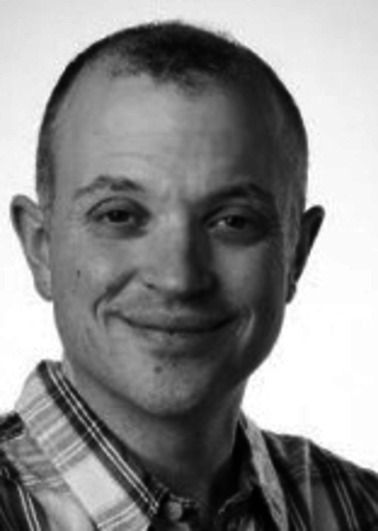# The Chemical Recycling of Polyesters for a Circular Plastics Economy: Challenges and Emerging Opportunities

**DOI:** 10.1002/cssc.202100400

**Published:** 2021-05-05

**Authors:** Jack Payne, Matthew D. Jones

**Affiliations:** ^1^ Centre for Sustainable and Circular Technologies University of Bath Claverton Down Bath BA2 7AY UK; ^2^ Department of Chemistry University of Bath Claverton Down Bath BA2 7AY UK

**Keywords:** catalysis, chemical recycling, circular economy, polyesters, upcycling

## Abstract

Whilst plastics have played an instrumental role in human development, growing environmental concerns have led to increasing public scrutiny and demands for outright bans. This has stimulated considerable research into renewable alternatives, and more recently, the development of alternative waste management strategies. Herein, the aim was to highlight recent developments in the catalytic chemical recycling of two commercial polyesters, namely poly(lactic acid) (PLA) and poly(ethylene terephthalate) (PET). The concept of chemical recycling is first introduced, and associated opportunities/challenges are discussed within the context of the governing depolymerisation thermodynamics. Chemical recycling methods for PLA and PET are then discussed, with a particular focus on upcycling and the use of metal‐based catalysts. Finally, the attention shifts to the emergence of new materials with the potential to modernise the plastics economy. Emerging opportunities and challenges are discussed within the context of industrial feasibility.

## Graphical abstract

**Reimagine a future with plastics**: In this Review, catalytic chemical recycling strategies for poly(lactic acid) (PLA) and poly(ethylene terephthalate) (PET) are presented, with a particular focus on upcycling and the use of metal‐based catalysts. Recent developments are critically discussed within the context of industrial feasibility.

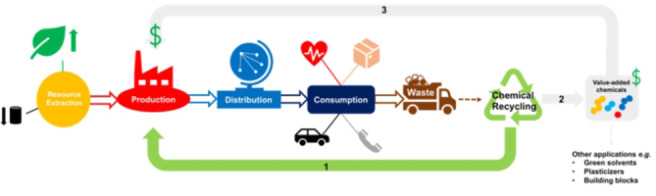

## 1 Introduction

**Figure 1:**
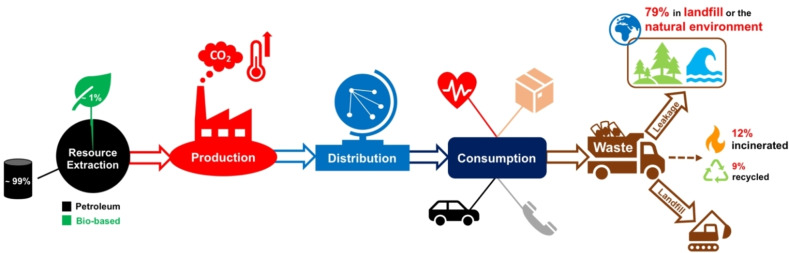
Linear model of a petroleum‐based plastics economy.

Plastics have played a crucial role in human development since their commercialisation in the 20th century, revolutionising key sectors such as transport, communications and healthcare.^[1]^ Whilst their inherent strength and durability is revered during their functional lifetime, such properties render plastics a pervasive environmental pollutant at end‐of‐life. The industries reliance on a depleting fossil feedstock, coupled with a linear model, serves to confound mounting environmental concerns (Figure 1).[^1^, ^2^, ^3^, ^4^, ^5^] It has been estimated of the 8.3 billion tonnes of plastic manufactured between 1950 to 2015, 6.3 billion tonnes is now waste, with 79 % accumulating in either landfill or the natural environment.^[6]^ Whilst prevalent on land, ocean plastics exemplify current levels of plastic pollution within the environment.[^7^, ^8^] In 2018, it was reported the Great Pacific Garbage Patch (GPGP) consisted of approximately 1.8 trillion plastic fragments, collectively weighing 79000 tonnes, and continues to grow annually.^[8]^ It is therefore unsurprising recent initiatives have emerged proposing plastics be banned outright, perhaps most notably in packaging applications, and replaced by alternative materials such as paper, glass and aluminium. However, in the face of increasing public scrutiny, it is imperative research continues to underpin informed decisions to avoid unintended environmental consequences. Indeed, despite being traditionally perceived as less environmentally friendly, life cycle analysis (LCA) has shown a poly(ethylene terephthalate) (PET) bottle to be significantly less carbon intensive relative to its glass and aluminium counterpart.^[9]^ Moreover, the social and economic value of plastics is often overlooked. In Europe, the plastics economy comprised close to 60000 companies, supporting 1.6 million jobs and turning over €360 billion in 2018.^[10]^ Consequently, a solution to the plastics dilemma is rather more complex than an outright plastic ban. A complete system redesign of the economy is required to mitigate anthropogenic activity and ensure its long‐term future. It is clear feedstock selection requires urgent revision with petroleum‐based products accounting for approximately 99 % of all processed plastics, consuming approximately 6 % of oil produced globally, which is projected to increase to 20 % by 2050.[^2^, ^11^] Bio‐based plastics represent a promising solution, but market penetration remains low (<1 %) due to a high production cost and inferior performance, for some applications, relative to conventional synthetic plastics.[^3^, ^12^] Nonetheless, it is anticipated increasing public awareness, coupled with legislation and a high oil price, will drive the uptake of bio‐based products. However, the plastic industry is characterised by a high product turnover, owing to an anticipated life expectancy of typically less than 1 year.[^2^, ^11^] Indeed, 1 million plastic bottles are produced per minute, with single‐use plastics equating for 47 % of the waste stream.[^2^, ^13^] Consequently, in pursuit of a sustainable plastics economy, utilisation of a renewable feedstock is not the answer unless it is complemented by comprehensive waste management strategies. This necessitates sufficient collection and sorting infrastructure to manage the large quantities of waste produced and minimise leakage. However, 32 % of plastic packaging waste escapes current collection systems, whilst emerging economies have little to no infrastructure.[^2^, ^5^, ^7^] Therefore, the waste crisis can be expected to worsen in the absence of positive, proactive intervention as plastics remain in the growth phase, with use expected to double within the next 20 years and production projected to exceed 1 billion tonnes per year by 2050.[^2^, ^14^] Presently, 40 % of post‐consumer plastic waste (PCW) is destined for landfill, where non‐biodegradable plastics can persist for decades.[^1^, ^2^, ^5^] Whilst immediate environmental impact is limited to land use and collection/transport, obvious benefits include potential greenhouse gas (GHG) sequestration and targeted waste depositing. Alternatively, industrial composting can facilitate the degradation of biodegradable plastics, such as poly(lactic acid) (PLA), limiting their environmental impact.^[5]^ However, both methods align with a linear model and fail to capture embedded material value. Whilst incineration represents a possible waste valorisation strategy, consuming 14 % of PCW, comprehensive LCAs favour recycling both in terms of energy use and GHG production.[^15^, ^16^] Thus, it is clear recycling will play a pivotal role in facilitating the industries transition to a bio‐based circular model, one concerned with material recapture and reuse.[^1^, ^2^, ^5^] Mechanical recycling is extensively exploited in the reprocessing of plastic packaging, accounting almost entirely for Europe's (EU 28+2) average packaging recycling rate, which equated to 42 % in 2018.^[10]^ However, the process is limited by eventual material downcycling, owing to thermomechanical degradation facilitated by the harsh remelting conditions used.^[5]^ Plastic oxidation over their functional lifetime increases their susceptibility to such detrimental side reactions during reprocessing.^[17]^ Consequently, there is an industry appetite to diversify the existing portfolio of plastic waste management strategies, with a particular focus on preserving, or indeed upcycling, waste material market value. A potential solution to this is chemical recycling, which will form the primary focus of this Review.

Recently, there have been a number of comprehensive polymer recycling Reviews published.[^5^, ^17^, ^18^, ^19^, ^20^, ^21^, ^22^] However, with the field rapidly expanding, numerous advancements have been made in recent years. Herein, we aim to highlight recent developments in the sustainable chemical recycling of two commercial polyesters, namely PLA and PET. The concept of chemical recycling will first be introduced, highlighting challenges and opportunities within the context of depolymerisation thermodynamics. Chemical recycling methods for PLA and PET will then be discussed, with a particular focus on upcycling and the use of metal‐based catalysts. We do not intend this to be an exhaustive account but instead endeavour to highlight key contributions and contextualize their impact. Emerging opportunities and challenges within the field are discussed within the context of industrial feasibility.

## 2 Chemical Recycling of Plastics

### 2.1 Principle of chemical recycling

The chemical recycling of plastic waste exploits a chemical transformation (e. g., hydrolysis, transesterification, hydrosilylation, etc.) to either recapture virgin monomer (closed‐loop) or directly convert it into other useful synthetic chemicals/feedstocks (open‐loop). Central to this concept is the polymer backbone bearing functionality susceptible to cleavage, for example, ester linkages found in polyesters. Potential benefits relative to mechanical recycling include:

1. removes material downcycling, thus promoting the long‐term retention of material value within the plastics economy
2. potential for upcycling plastic waste, enabling value‐added chemicals to be accessed for enhanced economic performance
3. access raw virgin feedstocks, such as lactic acid from PLA, whilst preserving product quality.[^5^, ^17^, ^22^, ^23^, ^24^]

### 2.2 Depolymerisation energetics

**Figure 2:**
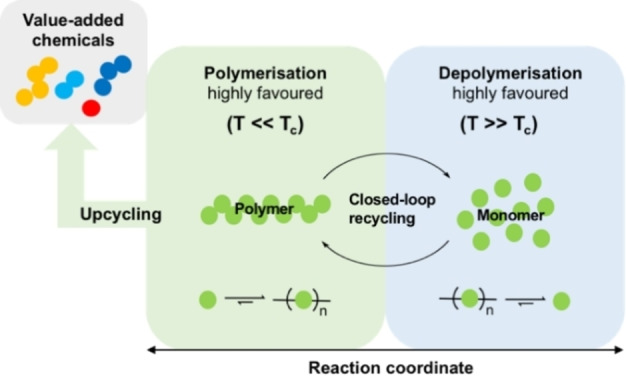
Overview of chemical recycling from an energetic perspective, considering closed‐loop recycling and upcycling.

In order to adopt a systemic approach to depolymerisation, it is important to first consider the fundamental thermodynamic and kinetic principles governing polymerisation.[^17^, ^22^, ^25^] Traditionally, exergonic polymerisations (Δ*G*_p_<0) are driven by a large exothermic enthalpic (Δ*H*_p_) driving force.[^5^, ^26^] Intuitively, this must dominate an entropic (Δ*S*_p_) forfeit conceded due to a reduction in degrees of freedom as monomer is consumed. At polymerisation equilibrium, the change in Gibbs free energy (Δ*G*_p_) is zero, and thus a critical temperature (*T*_c_) can be described exclusively as a function of Δ*H*_p_/Δ*S*_p_. Traditionally, Δ*H*_p_ and Δ*S*_p_ are negative and *T*_c_ is termed the ceiling temperature.[^17^, ^27^] Systems that favour polymerisation below *T*_c_ and depolymerisation above *T*_c_ will form the basis of this Review (Figure 2). Industrially relevant polymer (*M*_n_>10000 g mol^−1^) can be produced by careful consideration of the reaction conditions used in alignment with the Carother's equation and Le Châtelier's principle.[^17^, ^28^] It is thus clear the magnitude of Δ*H*_p_/Δ*S*_p_ dictates the temperature difference between complete polymerisation and the reverse process; depolymerisation.

However, polymer composition has significant ramifications on *T*_c_ and therefore their amenability to chemical recycling. Polyolefins consist of inherently inert sp^3^‐hybridised C−C and C−H bonds and thus require harsh conditions (250–400 °C) to overcome high activation barriers (*E*_a_=150–300 kJ mol^−1^) associated with pyrolysis.[^17^, ^29^] However, preceding catalytic pyrolysis methods utilising temperatures ≥500 °C have been reported.[^22^, ^30^, ^31^, ^32^] Product selectivity is also problematic, generally characterised by downgrading to fuels and waxes of varying chain length and saturation, owing to a homogenous polymer backbone.[^22^, ^30^, ^31^, ^32^, ^33^, ^34^] High‐density poly(ethylene) (HDPE) offers minimal monomer recovery (22–25 %), whilst high monomer yields (up to 94 %) have been reported for poly(propylene) (PP).[^17^, ^35^, ^36^] Such methods are practical from a plastic accumulation perspective and offer some net energy/material recovery. However, their high energy intensity releases damaging CO_2_ emissions into the environment. Thus, it is clear the development of selective and mild recycling strategies for polyolefins remains a prevalent challenge in the field. However, extreme exergonicity renders chemical recycling unsuitable, though this is not to say alternative strategies should not be aggressively pursued to mitigate plastic pollution. Indeed, polyolefins accounted for almost two‐thirds of global plastic production in 2015.^[11]^ Since such materials are not the primary focus of this report, we direct the interested reader to two excellent Reviews that highlight recent developments within the field.[^17^, ^22^]

Polyolefins represent an exergonic threshold, with chemical recycling lending itself to exergonicities approaching neutrality. Coates and Getzler recently described the ideal system as one that exhibits sufficient polymerisation exergonicity as to achieve high conversion and molecular weights rapidly, whilst retaining high selectivity under mild conditions. These features should be reflected just above *T*_c_ in the corresponding depolymerisation process.^[17]^ However, polymers can become kinetically trapped in the absence of a reactive chain‐end due to end‐capping, which can be as simple as a proton. This increases the thermal stability of the polymer well beyond its *T*_c_, necessitating thermodynamic and kinetic driving forces be considered in tandem during polymer design.^[17]^ A sustainable plastics economy relies on leveraging the intricate balance between polymerisation and depolymerisation energetics to deliver a truly sustainable and circular product portfolio. However, the current waste crisis poses an interesting dilemma: should research focus on developing recycling strategies compatible with existing products or favour a complete system redesign? We argue both avenues should be pursued in parallel to ensure future growth endeavours to address existing challenges, whilst anticipating future needs and concerns.

### 2.3 Catalysis

Catalysis will undoubtedly play a crucial role in ensuring the commercial viability of chemical recycling by improving reaction efficiency and reducing waste. Indeed, catalysis is exploited in approximately 90 % of industrial chemical processes and contributes over £50 billion to the UK economy annually.^[37]^ Whilst pyrolysis is highly material dependent, catalysis offers the opportunity to precisely engineer the process conditions used and products manufactured. An excellent example of metal‐based catalysis underpinning commercial viability is that of Ziegler‐Natta applied to olefin polymerisation. Such catalysts enabled commercialisation of the process in 1954, ushering in an era of unprecedented economic and academic investment in order to realise the tangible societal benefits of plastics.[^17^, ^38^] For example, plastic components lower the environmental impact of vehicles by a factor of 4, whilst plastic insulation saves 250 times the energy used for its production.^[39]^ Whilst this has led to significant developments in the field of polymerisation catalysis, our attention must now be diverted towards depolymerisation in equal measure to mitigate plastic pollution and ensure the plastic economy's long‐term future. Before considering plastics amenable to chemical recycling, focusing on polyesters and the application of metal‐based catalysts, we will first consider societal and economic challenges associated with the uptake of such technology.

### 2.4 Society, infrastructure and economics

Chemical recycling has long been an established technology with commercial examples including the PETCORE system, the Eastman Chemical Company (EEC) method and the DuPont process.^[40]^ However, such processes are sensitive to feed impurities, requiring a pre‐treatment step. This coupled with high capital expenditure (CAPEX) and process costs relative to cheap petrochemical feedstock has limited their widespread application. Common waste stream contaminants include foreign debris and other plastics arising due to sorting mistakes or in instances when separation is difficult to achieve (for example PE and PP).^[22]^ Poly(vinyl chloride) (PVC) is particularly problematic due to its propensity to eliminate HCl upon heat treatment, which can lead to reactor corrosion, precluding mechanical recycling.^[41]^ Indeed, PVC contamination as low as 100 ppm has previously been reported to adversely impact the quality of the recycled product.^[42]^ Plastics are also inherently heterogeneous, containing numerous additives (e. g., plasticizers, stabilizers and pigments) for performance and aesthetic purposes.^[43]^ Beyond the manufacturer, their identity is often unknown due to intellectual property (IP) rights. Consequently, if treated in isolation with respect to product commercialisation, their potentially detrimental impact on polymer recyclability remains unknown until end‐of‐life, at which point it is too late. Moving forward, industry/consumers may need to concede on product expectations when additives are used solely for aesthetic purposes (e. g., pigments in carbonated drinks bottles) unless green alternatives that uphold recyclability can be developed. Education will play a key role in reducing resistance to such change and promoting consumer engagement. Multicomponent and composite plastics serve to confound the aforementioned challenges.^[22]^ We therefore identify a clear opportunity to collaborate fruitfully with industry to deliver transferable research and avoid such pitfalls. For emerging materials, this necessitates embedding recyclability at the design phase whilst maintaining a competitive cost‐to‐performance ratio.

However, despite a clear industry appetite for robust and selective recycling strategies, a serious imbalance remains between waste generation and recovery.[^5^, ^22^] This can be attributed to both a lack of infrastructure (e. g., collection and sorting) and insufficient waste management portfolio. Indeed, only 14 % of plastic packaging collected is intended for recycling, with closed‐loop (i. e., collected and reprocessed for the same application) accounting for just 2 %.[^2^, ^22^] It has been estimated for PET chemolysis facilities to be economically viable they require a minimum throughput of 1.5×10^5^ tonnes p/a.^[44]^ Significant capital investment will undoubtedly underpin realising this future, but industry has been cautious. Five recent signatories of the “The New Plastics Economy Global Commitment” pledged $200+million towards enabling a circular plastics economy.^[45]^ Whilst promising, this remains low relative to the projected $15–20 billion of CAPEX required annually to achieve a recovery rate of 50 % by 2030.^[46]^ Aggressive investment strategies can be incentivised through developing renewable products/processes that compete with, or indeed outperform, their petrochemical‐based counterpart. Industry must also adopt a mindset that values plastic waste as an untapped resource, which is anticipated to grow from 260 to 460 million tonnes between 2016–2030 based on current disposal rates.^[46]^ Moreover, recycled content demand is expected to exceed 5 million tonnes by 2025, equivalent to 25 million barrels of oil being left in the ground.^[45]^ Over the last decade, global petrochemical and plastic industry investment has totalled between $80–100 billion each year.^[46]^ If such funds can be directed towards enabling a sustainable and circular plastics economy, we remain optimistic of taking significant strides towards achieving 2025 targets.^[45]^ Legislation will also undoubtedly play a crucial role in ascertaining a circular plastics economy, whether it be through promoting the uptake of renewable technology (e. g., economic subsidies) or influencing consumer habits. Moreover, such policy need not be inherently complex to achieve significant disruption. For example, following the introduction of a simple 5p plastic bag charge in 2015, plastic bag sales reduced by 86 % between 2015–2018 among England's major supermarkets, removing over 9 billion plastic bags out of circulation.^[47]^ However, policy requires standardisation with regards to plastic disposal. In the UK, such policy can vary considerably between local and regional authorities owing to a certain degree of devolution, which generates discontinuity at a national level. This leads to consumer frustration and confusion, which encourages sorting mistakes and a tendency not to recycle. Efficient recycling strategies will only achieve their desired environmental impact if all components in the supply chain are connected and operate harmoniously.

Whilst we focus on the development of waste management strategies in this Review, this is not to say it is inherently any more, or less, important than any other individual component in the supply chain. It is imperative all components are developed in tandem to deliver an integrated plastics economy that practices circularity and sustainability. It is only by adopting this stance that meaningful change can be realised within the next decade and beyond.

### 2.5 Polyesters

**Figure 3:**
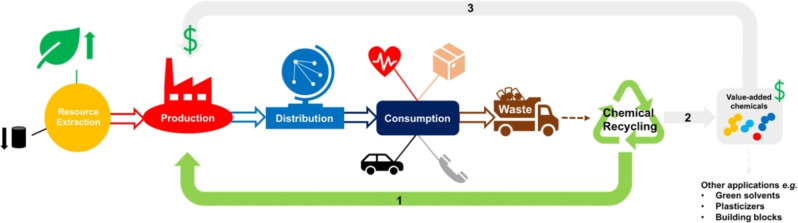
Diagram representing the potential for chemical recycling to introduce circularity into the plastics economy: (1) closed‐loop recycling or (2) transformation of plastic waste into value‐added chemicals that can be used in higher‐value applications or recirculated to access higher‐value plastics (3).

Polyesters represent ideal candidates for chemical recycling owing to the presence of a highly polar sp^2^‐hydridised carbonyl bond (C=O), which is susceptible to nucleophilic attack. It is therefore unsurprising most progress in catalytic chemical recycling pertains to polar plastics.^[22]^ Society's varied polyester use demands equally diverse recycling strategies, rendering a “one‐solution‐fits‐all” scenario unrealistic. We encourage the scientific community to exploit the inherently vibrant and diverse field of carbonyl chemistry in system and product design.^[48]^ It is envisaged an indefinite chemical recycling closed‐loop will increase recycled content in today's products, reducing society's dependence on depleting fossil reserves, whilst promoting the uptake of bio‐based alternatives. However, it is the potential to access higher‐value chemicals for use in both the plastic industry and beyond that creates a unique differentiating value proposition relative to other waste management strategies (Figure 3). This will be particularly adventitious for plastics where recovering the monomer may be economically unviable. Recently, a number of promising advancements have been made, although numerous key challenges remain. For research to be considered industrially relevant, it must fulfil the following criteria:

1. high process efficiency under mild conditions
2. high product selectivity in the presence of mixed plastics
3. robust catalysts tolerant to common plastic waste stream contaminants, including additives and debris
4. simple catalyst recovery and reuse, maintaining performance between cycles
5. metal‐based systems should exploit the use of cheap and earth‐abundant metals in combination with scalable ligands.

The aforementioned criteria will provide the framework by which recent developments in the field will be assessed. The plastic waste crisis demands the development of recycling strategies for both emerging and established plastics in parallel. We adopt this approach in this Review, first considering developments for PLA, an emerging bio‐based plastic, before discussing those pertaining to PET, an established polyester with a significantly higher market share.^[11]^

## 3 Chemical Recycling of Poly(lactic acid)

### 3.1 Poly(lactic acid)

**Figure 4:**
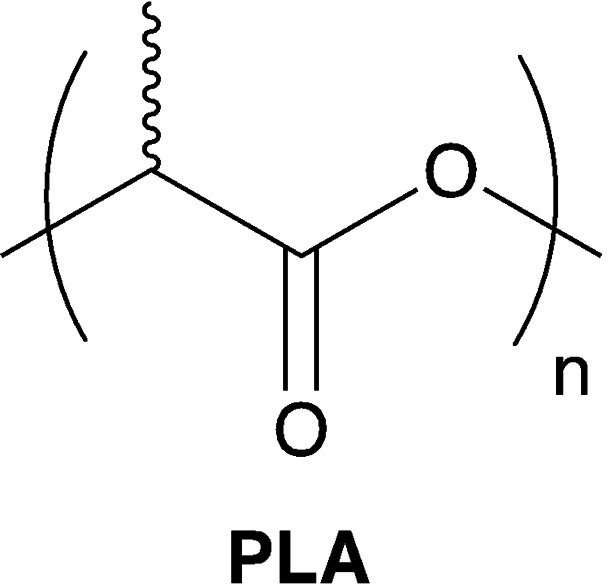
Polymeric structure of PLA.

PLA is a renewable and biodegradable aliphatic polyester based on a repeating lactic acid monomer (Figure 4), sourced from the microbial fermentation of starch‐rich feedstocks, such as corn and sugar.[^5^, ^49^, ^50^] Industrially, PLA is produced from the ring‐opening polymerisation (ROP) of l *‐*lactide under solvent‐free conditions. This method exploits a Sn(Oct)_2_ (Oct=2‐ethylhexanoate) catalyst operating via a coordination–insertion mechanism to produce poly(l‐lactic acid) (PLLA) of high and well‐defined *M*_n_.^[51]^ Toxicity concerns associated with the industry standard [Sn(Oct)_2_] has stimulated considerable research into sustainable and biocompatible alternatives.[^51^, ^52^, ^53^, ^54^] This remains an active area of research although it falls beyond the scope of this Review and thus will not be discussed further. PLA has been the subject of intense academic interest over the last 20 years owing to its green credentials.[^5^, ^11^, ^55^, ^56^, ^57^, ^58^, ^59^] PLA possesses intrinsic biocompatibility and thus has been widely exploited in the biomedical industry. Common applications include use in tissue scaffolds, sutures and drug delivery systems.[^5^, ^60^, ^61^] PLA has also found use in food and packaging material applications.[^5^, ^11^, ^55^, ^56^, ^57^, ^58^, ^60^]

Despite being a commercially available polymer, its widespread use has been limited by a high production cost relative to traditional synthetic plastics. This can be attributed to complexity associated with the fermentation and purification of lactic acid, accounting for approximately 50 % of total production costs.^[62]^ It is therefore unsurprising PLA accounted for just 13.9 % of bioplastic production in 2019.^[63]^ Thus, research has been devoted to reducing production costs by targeting the production of lactide directly in one‐step processes, exploiting the use of shape‐selective catalysis and gas‐phase reactions.[^64^, ^65^, ^66^, ^67^, ^68^, ^69^, ^70^] However, it is clear PLA will play a prominent role in a future plastics economy as the uptake of bio‐based products increases.^[5]^ Indeed, the production and use of PLA has the potential to reduce GHG emissions and non‐renewable energy use by 40 and 25 %, respectively, compared to traditional petroleum‐based plastics, including PE and PET.[^3^, ^5^, ^71^, ^72^] In 2018, Total Corbion constructed a new 75000 tonne p/a plant in Thailand, signifying market growth.^[73]^

However, despite its green credentials, PLA waste is a potential contributor to plastic pollution if irresponsibly handled at end‐of‐life. PLA is often praised as a biodegradable alternative, although this leads to the misconception that it readily degrades in the natural environment.^[74]^ PLA biodegrades efficiently into CO_2_ and H_2_O under industrial composting conditions, requiring elevated temperatures (60 °C) and high relative humidity in the presence of thermophilic microbes.[^5^, ^75^, ^76^, ^77^, ^78^, ^79^] Complete biodegradation has been reported within 30 days under such conditions.[^75^, ^79^] Conversely, degradation can take up to a year in domestic composters at 20 °C, which can be reduced to 12 weeks above 25 °C.[^80^, ^81^] PLA's tendency to persist in the marine environment raises further concerns. Recent studies observed no degradation within 1 year under laboratory conditions simulating static seawater, although weight loss was noted under dynamic conditions via mechanical processes.[^82^, ^83^, ^84^, ^85^]

In light of such challenges, there is a clear need to develop sustainable chemical recycling strategies to assist incorporation of PLA into the circular economy. Given PLA's relatively low, but increasing, market share at present, this represents a unique opportunity to potentially introduce large‐scale commercialisation and complementary recycling methods in parallel. This would assist plastic pollution mitigation from the outset, whilst providing a model framework for future product design/deployment.

### 3.2 Hydrolysis to lactic acid

**Figure 5:**
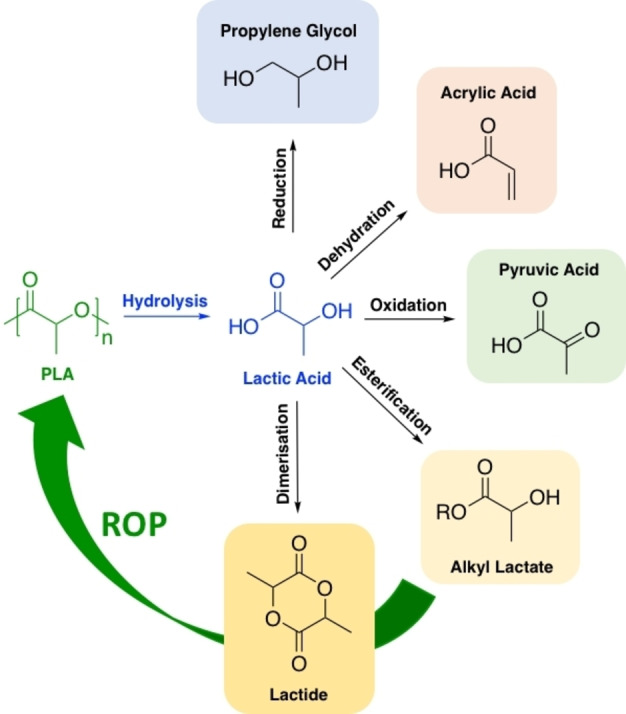
Hydrolysis of PLA to lactic acid with examples of further transformations to value‐added commodity chemicals. Similar transformations are possible starting from alkyl lactates, whilst the green arrow highlights possible circularity (via lactide) in the PLA supply chain.^[89]^

PLA hydrolysis produces lactic acid, which has been identified as a future platform chemical for the production of a wide range of value‐added commodity chemicals (Figure 5).[^5^, ^86^, ^87^] Current lactic acid production capacity is approximately 400000 tonnes p/a, which is projected to increase annually by 5–8 %.[^5^, ^18^, ^88^] Lactic acid is envisaged to play a crucial role in ascertaining a low‐carbon future, underpinned by a bio‐based circular economy. Consequently, considerable research has been devoted to PLA hydrolysis.

PLA hydrolysis is known to proceed via two possible mechanisms, dictated by the rate of water diffusion relative to bond breaking. This is dependent on a number of parameters including molecular weight, pH and temperature. Homogeneous sample mass loss dominates when water diffusivity is high, whilst heterogeneous surface erosion is observed when water diffusivity is low.[^89^, ^90^] McKeown and Jones^[89]^ recently published a detailed account of PLA hydrolysis, particularly from a mechanistic perspective. Here we do not intend to reproduce such work but instead highlight key contributions.

Pioneering work by Tsuji et al.[^91^, ^92^, ^93^, ^94^, ^95^, ^96^, ^97^] details early developments within the field. Initial work considered a 5 wt % solution of PLLA (*M*_n_=170000 g mol^−1^) between 180–350 °C.^[91]^ An optimum hydrolysis temperature of 250 °C was found, achieving 90 % l *‐*lactic acid yield within 20 min (*E*_a_=51.0 kJ mol^−1^). Above 250 °C, racemisation became more prevalent, culminating in lactic acid decomposition into CO_2_, CO and CH_4_ at 350 °C. Degradation via a homogenous mass loss mechanism was found to proceed independent of reaction temperature (120–250 °C) and PLA phase (melt or solid).^[92]^ Such high reaction temperatures are characteristic of PLA hydrolysis owing to its inherent insolubility in the reaction media, rendering the process energy intensive. The effect of average block length on the degradation of stereoblock PLA has also been investigated.^[94]^ Rapid degradation of atactic segments was observed, whilst a decrease in hydrolysis rate was noted for increasing stereoblock length. Hirao and Ohara^[98]^ have demonstrated the application of microwave heating to achieve enhanced hydrolysis rates. Using a relatively concentrated solution of PLA (75 wt %, *M*_n_=96000 g mol^−1^), maximum lactic acid yield was achieved within 800 min at 170 °C, which could be reduced to 120 min under microwave irradiation. However, this process is limited to 45 % lactic acid yield before racemisation reduces optical purity of the final product.

Piemonte and Gironi[^99^, ^100^] have contributed substantially to the field from a kinetic perspective. Recent studies have investigated hydrolysis between 140–180 °C for varying concentrations of PLA (5–50 wt %), observing 95 % conversion to lactic acid within 120 min between 160–180 °C. The kinetic reaction rate was found to be independent of PLA concentration and characterised by two distinct reaction mechanisms: (1) a two‐phase reaction (*E*_a_=53.2 kJ mol^−1^) and (2) an autocatalytic effect (*E*_a_=36.9 kJ mol^−1^). This autocatalytic effect had previously been reported by Siparsky et al.^[101]^ and arises due to an increase in the number of carboxylic acid end groups as hydrolysis proceeds, which decreases the pH of the solution. Villani and co‐workers^[102]^ have subsequently extended this kinetic model to higher reaction temperatures (170–200 °C), achieving complete PLA conversion within 90 min.

Given the challenge of solubilising PLA in H_2_O, water/ethanol mixtures (50 % ethanol) between 40–90 °C have recently been reported.[^103^, ^104^] The presence of ethanol causes the polymer to swell, facilitating enhanced water diffusivity, which reduces the activation barrier [*E*_a_(H_2_O)=101.4 kJ mol^−1^, *E*_a_(H_2_O/EtOH)=93.4 kJ mol^−1^]. It was predicted oligomers suitable for repolymerisation could be obtained after 29 h at 90 °C, whilst prolonged reaction would achieve 95 % yield of lactic acid after approximately 41 h. Whilst such conditions are considerably less energy intensive relative to traditional hydrolysis systems, such reactions times are unreasonable at an industrial scale.

To overcome this challenge, commercial processes typically use a strong inorganic acid (H_2_SO_4_, HNO_3_) or base [NaOH, Ca(OH_2_)_2_] catalyst.[^5^, ^105^] A patented example is described by Coszach et al.^[106]^ who demonstrated PLA hydrolysis in both the presence and absence of NaOH, the latter being particularly commercially adventitious since it removes the need for harsh and highly corrosive reagents. The hydrolysis process proceeded between 80 and 180 °C with pressures of up to 10 bar. In the absence of catalyst, reaction temperatures can be as high as 350 °C.^[89]^ Unsurprisingly, to the best of our knowledge, no examples of PLA hydrolysis mediated by a discrete metal‐based catalyst have been reported. This is presumably due to their sensitivity to hydrolytic degradation, highlighting the need for robust metal‐based catalysts in pursuit of sustainable PLA hydrolysis. Song et al.^[107]^ have demonstrated the use of ionic liquids (ILs) for the relatively mild hydrolysis of PLA. [Bmim][OAc] was identified as the outstanding candidate, achieving up to 94 % lactic acid yield within 2 h at 130 °C (*E*_a_=133.9 kJ mol^−1^). The product was recovered by addition of calcium carbonate to precipitate calcium lactate in good yield (up to 76 %). Promisingly, [Bmim][OAc] could be recycled seven times with no decrease in performance. However, this system is limited by a high catalyst loading (50 wt % based on PLA), which is unscalable based on catalyst cost (Sigma Aldrich, 100 g, £200).

Enzymatic processes have also previously been reported.[^5^, ^108^, ^109^, ^110^, ^111^] Whilst their industrial feasibility is hindered by possible scalability issues, it is clear biocatalysis will play an increasingly important role in enabling the bioeconomy.[^112^, ^113^, ^114^]

### 3.3 Transesterification to alkyl lactates

**Scheme 1:**
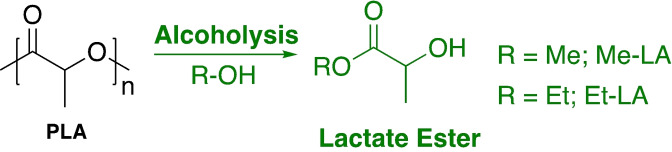
Metal‐mediated alcoholysis of PLA to afford lactate esters, otherwise referred to as alkyl lactates.^[89]^

Whilst the depolymerisation of PLA to lactic acid is one circular economy approach, perhaps a more attractive option is the direct transformation of waste feedstock into value‐added chemicals. Consequently, the transesterification of PLA into alkyl lactates (lactate esters) has received increasing attention (Scheme 1). Low‐molecular lactate esters have been identified as potential green solvent replacements for traditional petrochemical‐based solvents owing to their inherent biodegradability and low toxicity. Moreover, their low vapour pressure ensures they are safer and easier to handle than conventional solvents. As such, lactate esters lend themselves to a diverse range of sectors, including the pharmaceuticals, agriculture and polymer industry.[^5^, ^115^, ^116^] There is also the potential to realise enhanced economic performance through waste upcycling, a particularly attractive quality to industry. The Et‐LA market is estimated to reach $92 million by 2024 and currently trades at £2.54–3.49 per kg relative to £1.69 per kg for virgin PLA.[^42^, ^117^] Traditionally, such materials are resource and energy intensive to produce, providing significant scope for process optimisation in accordance to the 12 principles of green chemistry.[^5^, ^118^] Recently, the metal‐mediated alcoholysis of lactide has been shown to be an effective alternative.[^119^, ^120^, ^121^] However, this method is arguably an inefficient use of a direct PLA precursor and fails to utilise the PLA waste stream. We will therefore focus on PLA transesterification methods.

Numerous patented processes have been reported for PLA alcoholysis, detailing the use of a range of solid acid/base catalysts (CaO, Montmorillonite K10, Nafion‐H) and solvents (ILs, toluene, lactate esters, chloroform, alcohols).[^89^, ^105^] DuPont possesses a patent for PLA degradation into various lactate esters (R=Me, Et and *n*Bu) in the presence of H_2_SO_4_, achieving high conversion (69–87 %) within 2 h between 150 and 190 °C.^[122]^ However, the acid catalyst used is both highly corrosive and toxic, and thus effort in the literature has focused on developing more environmentally friendly alternatives.

**Figure 6:**
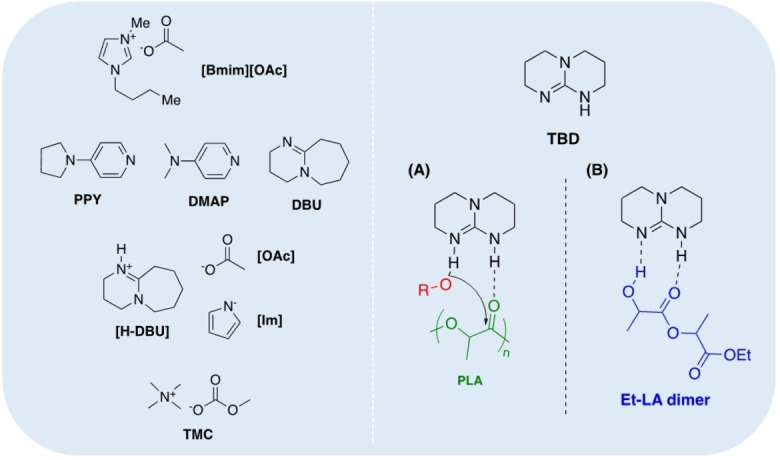
Selected examples of organocatalysts reported for PLA transesterification. (A) Proposed dual‐activation transesterification mechanism for TBD and (B) reaction inhibition by intramolecular binding of lactate dimer to TBD.

To this end, Song et al.^[123]^ reported the first example of PLA (*M*_w_=400000 g mol^−1^) methanolysis mediated by a range of ILs (Figure 6). [Bmim][OAc] was identified as the outstanding candidate, consistent with PLA hydrolysis, achieving up to 93 % Me‐LA yield within 3 h at 115 °C (*E*_a_=38.3 kJ mol^−1^). [Bmim][OAc] could be recycled 6 times without a reduction in activity, although a high loading was noted (50 wt % based on PLA). The use of ILs in combination with simple metal salts [e. g., Zn(OAc)_2_ and FeCl_3_] has also been shown to facilitate PLA degradation under milder conditions.[^124^, ^125^] For example, 2[Bmim][OAc]‐Zn(OAc)_2_ achieved 92 % Me‐LA yield within 2 h at 110 °C, consistent with a lower activation energy (*E*_a_=21.0 kJ mol^−1^). This synergistic reactivity enhancement can likely be attributed to greater C=O activation in the presence of Lewis acid metals, facilitated by enhanced PLA dissolution. Despite ILs exhibiting superior activity and easier product separation relative to H_2_SO_4_, their scalability remains limited by their high cost and intrinsic viscosity.

Organocatalysts have also been reported for PLA transesterification (Figure 6).^[126]^ Hedrick and co‐workers^[127]^ have demonstrated the use of 4‐pyrrolidinopyridine (PPY) and 4‐dimethylaminopyridine (DMAP) for the alcoholysis of PLA, focusing on controlled degradation to target molecular weights. Recently, Enthaler and co‐workers^[128]^ extended the use of DMAP for PLA methanolysis under microwave irradiation, achieving high Me‐LA yield within 10 min at 180 °C. The use of MeOH in a large excess (23.1 equiv.) allows the reaction to proceed under neat conditions, negating the need for potentially harmful solvents that are typically a significant source of waste in industry.^[5]^ Moreover, this simple catalytic system exhibited reasonable tolerance to plastic contaminants and additives for PLA sourced from 16 commodity applications. High activity was retained on substituting DMAP for 1,8‐diazabicyclo[5.4.0]undec‐7‐ene (DBU). Liu et al.^[129]^ recently reported DBU‐based protic ILs for PLA (*M*_w_=400000 g mol^−1^) alcoholysis. Preliminary screening found [H‐DBU][OAc] offered the highest Me‐LA yield, achieving 91 % within 5 h at 100 °C. High lactate ester yields (76–89 %) were retained for higher‐chain alcohols under comparable conditions. Substitution of the anion for an imidazole‐based derivative afforded [H‐DBU][Im], capable of achieving 87 % Me‐LA yield within 1 h at 70 °C.^[130]^ This remarkable activity enhancement enabled polymer scope to be expanded to PET and poly(bisphenol A) carbonate (BPA‐PC), demonstrating catalyst versatility. McKeown et al.^[131]^ recently reported tetramethylammonium methyl carbonate (TMC) as a simple and cheap organocatalyst for versatile polymer degradation including PET, BPA‐PC and poly(*ϵ‐*caprolactone) (PCL). Promisingly, 100 % Me‐LA yield could be achieved within 1 h at 50 °C in THF. High activity was retained down to reasonably low catalyst loadings (0.5 mol %), which is commonly a limiting feature among organocatalysts, perhaps most notably in ILs. Leibfarth et al.^[132]^ have demonstrated 1,5,7‐triazabicyclo[4.4.0] dec‐5‐ene (TBD) to be an extremely efficient catalyst for PLA degradation. Indeed, TBD exhibited extremely high activity, achieving >90 % Et‐LA yield within 3 min at room temperature, which could be extended to a range of primary alcohols including MeOH, BuOH and BnOH. TBD's remarkable activity can likely be attributed to a dual‐activation mechanism, characterised by simultaneous activation of both the carbonyl group and incoming alcohol via H‐bonding (Figure 6a). Interestingly, transesterification of the ethyl lactate dimer proceeded significantly slower relative to bulk PLA (*M*_n_=76700 g mol^−1^). This retardation event was attributed to the formation of an intramolecular complex between the dimer and TBD, which subsequently inhibits activation of a further ethanol molecule (Figure 6b). An enantiomeric excess (*ee*) of >95 % confirmed preservation of stereochemistry in the lactate product from PLLA.

**Scheme 2:**
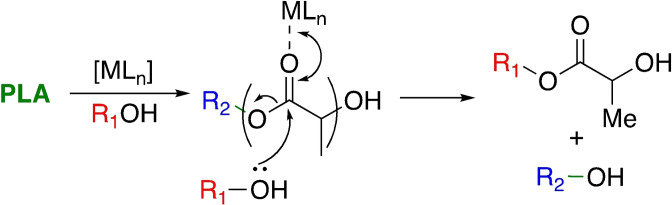
General metal‐mediated degradation mechanism of PLA into a lactate ester via transesterification with an alcohol, where R_1_ and R_2_ denote the alcohol chain length and growth polymer chain, respectively.

Despite product racemisation risking potentially costly and complex product separation, retention of stereochemistry in the final lactate product often remains overlooked in the literature. Whilst TBD clearly represents the benchmark for PLA alcoholysis from an activity standpoint, with degradation under ambient conditions adventitious both economically and environmentally, TBD remains limited by properties akin to H_2_SO_4_. Moreover, this system utilises CH_2_Cl_2_, a possible carcinogenic solvent, and thus is limited in practice relative to the 12 principles of green chemistry.^[118]^ A possible solution to this is metal‐mediated degradation (Scheme 2), although literature examples remain scarce despite the plethora of initiators reported for lactide polymerisation.[^51^, ^52^, ^54^]

The first example of metal‐mediated PLA alcoholysis dates back to 1945, concerning the use of ZnCl_2_ with temperatures up to 150 °C.^[133]^ A range of studies using commercially available metal salts have since been reported. Sanchéz and Collinson^[134]^ reported a strategy using Zn(OAc)_2_ for the selective degradation of PLA into Me‐LA from a 1 : 1 mixture of PLA and PET. At the boiling point of methanol, 65 % Me‐LA yield was obtained after 15 h. Under these conditions, PET was found to be non‐reactive and insoluble, which enabled solid PET to be separated by filtration post‐reaction. The formation of Zn(lactate)_2_ was detected by IR spectroscopy, possibly implicating this as the active species. Liu et al.^[135]^ investigated the activity of a wide range of simple, commercially available salts including NaOAc, NaOH, NaOMe, Zn(Octanoate)_2_, AlCl_3_ and SnCl_4_ ⋅ 5H_2_O. FeCl_3_ was identified as the outstanding candidate, achieving 87 % conversion to Me‐LA within 4 h at 130 °C in the absence of solvent (*E*_a_=32.4 kJ mol^−1^). The catalyst could be reused 6 times without any appreciable loss in activity following recovery via distillation of the lactate product. This is particularly impressive given catalyst recovery is often a limiting industrial feature of homogeneous catalysis. Recently, Enthaler and co‐workers[^136^, ^137^, ^138^, ^139^] have extensively reported the use of simple metal salts for PLA alcoholysis under microwave irradiation. Commercially available alkali halide salts of the general formula MX, such as KF, LiCl and KBr, were found to be potent catalysts for PLA (*M*_n_=43600–150400 g mol^−1^) methanolysis between 140–160 °C. Indeed, KF was shown to facilitate high yields of Me‐LA within 10 min and could be reused up to three times.^[136]^ It is anticipated the potassium cation activates the carbonyl to nucleophilic attack, whilst the fluoride anion simultaneously assists proton transfer. Sn(Oct)_2_ has also been shown to facilitate methanolysis for various end‐of‐life sources of PLA (*M*_n_=43600–150400 g mol^−1^).^[137]^ High Me‐LA yields were achieved between 140–180 °C at low catalyst loadings (0.05–0.25 mol %), observing turnover frequencies (TOFs) up 36900 h^−1^ at 180 °C. The scalability of this process was demonstrated at a 50 g scale using a PLA cup. Interestingly, the amount of MeOH was found to significantly impact Me‐LA yield, observing a reduction from quantitative to negligible yield upon shifting from 15.4 to 11.6 equiv. Plichta et al.^[140]^ had previously reported the use of Sn(Oct)_2_ for the partial alcoholysis of high molecular weight PLA (*M*_w_ *=*217000 g mol^−1^) in the presence of protic reagents, such as diols, diacids and macromolecules, for the design of block copolymers. To address toxicity concerns associated with Sn(Oct)_2_, Enthaler and co‐workers explored the use of environmentally benign alternatives, including bismuth‐ and zinc‐based salts.[^138^, ^139^] Promisingly, TOFs up to 13800 and 45000 h^−1^ were observed for bismuth subsalicylate and Zn(OAc)_2_, respectively, at 180 °C using 0.1 mol % catalyst. However, in both instances a large excess of MeOH (67.5 equiv.) was required, which limits process scalability. A reoccurring theme of this group's work is to assess the impact of various sources of end‐of‐life PLA (e. g., cup, bottle, coloured lids, contaminants) and mixed plastic waste streams (e. g., PLA+PET, nylon‐6, PVC, BPA‐PC) on catalyst activity and selectivity, both of which are integral for ensuring industrial viability. For both bismuth subsalicylate and Zn(OAc)_2_, high Me‐LA yield (>99 %) was retained irrespective of PLA source.[^138^, ^139^] Conversely, Me‐LA yield was found to vary more significantly for the alkali halide and Sn(Oct)_2_ systems, observing moderate to high yields (43–128 %). Note yields greater than 100 % were observed in instances when the starting material was assumed to be 100 % PLA but contained a substantial number of additives by mass (e. g., black sushi box). Generally, high activity and selectivity was retained in the presence of mixed waste streams, observing the concomitant degradation of BPA‐PC with PLA, whilst nylon‐6 and PET remain intact.[^136^, ^137^, ^139^] Sobota and co‐workers^[141]^ have explored the use of cheap and abundant magnesium and calcium catalysts for the solvothermal alcoholysis of PLA (*M*_n_=64200–115700 g mol^−1^). Using metallic magnesium or Mg(*n*Bu)_2_, efficient alcoholysis was achieved at 200 °C within 1 h using a wide range of linear and branched alcohols. Ethanolysis was scaled up to 1.5 kg, noting retention of polymer stereochemistry in the lactate product, confirmed by polarimetry. High reaction temperatures were favoured to avoid the use of excess alcohol, despite reasonable Et‐LA yields (71–88 %) being attainable as low as 100 °C in the presence of 4–10 equiv. of ethanol. In the absence of catalyst, high‐temperature regimes (220–260 °C) were required in the presence of 4 equiv. of ethanol based on ester linkages. Such high temperatures are consistent with work by Hirao et al.^[142]^ for the ethanolysis and butanolysis of PLA (*M*_n_=96000 g mol^−1^), which required conventional heating up to 210 °C and a large excess of alcohol (10 equiv.), although enhanced reaction rates were observed under microwave irradiation. Commercially available alkali/alkaline metals (Li−K/Mg−Ba) and selected alkoxides [e. g., Na(OEt), K(OEt), Ca(OMe)_2_], in addition to organometallic/chloride zinc, tin and aluminium reagents, were also investigated.^[141]^ All reagents exhibited good activity, achieving between 64–91 % Et‐LA yield at 200 °C within 1 h under autogenous pressure. Interestingly, the formation of Ca(lactate)_2_ was observed for calcium‐mediated alcoholysis, consistent with work by Sanchéz and Collinson.^[134]^

**Figure 7:**
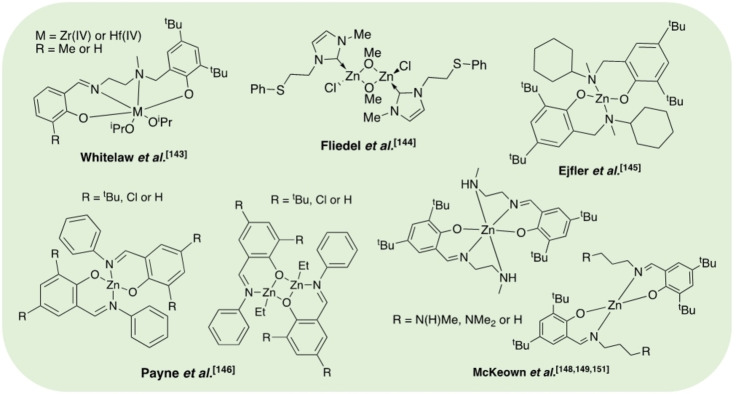
Discrete metal‐based catalysts reported for the transesterification of PLA.

**Scheme 3:**
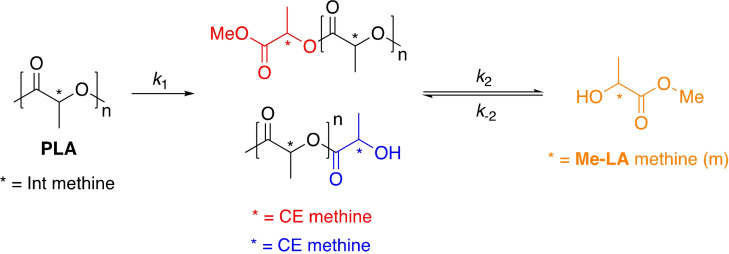
Two‐step reaction sequence for the production of Me‐LA from PLA via the intermediate formation of chain‐end groups. Consequently, the methine groups can be categorised as internal (int), chain‐end (CE) and those corresponding directly to the alkyl lactate (Me‐LA).^[149]^

Whilst such methods have the potential to overcome industry concerns associated with catalyst recovery and equipment corrosion, there is a clear opportunity to preserve activity under significantly milder conditions. This can likely be achieved through judicial choice of the metal‐ligand employed, although literature examples of discrete metal‐based complexes remain limited (Figure 7). Whitelaw et al.^[143]^ have previously reported a series of zirconium and hafnium(IV)‐salalen complexes for the production and degradation of PLA. It was proposed the addition of excess MeOH during post polymerisation work up facilitated the formation of a bismethoxide analogue, which appeared active for PLA methanolysis. The Hf^IV^‐salalen (R=Me) complex was found to degrade PLA samples of varying tacticities (atactic and isotactic; *M*_n_=10000–200000 g mol^−1^), achieving 75 % conversion to Me‐LA within 24 h at room temperature for a commercial PLLA source (*M*_n_=200000 g mol^−1^). However, ligand complexity limits the scalability of these systems, highlighting the need for facile ligand preparation. Zn^II^‐complexes are arguably the most studied for PLA recycling due to a strong literature precedent as highly active initiators for lactide polymerisation, coupled with zinc being an inexpensive and biocompatible metal.[^51^, ^52^, ^54^] Fliedel et al.^[144]^ reported the first example of a Zn^II^‐complex for the controlled degradation of PLA, namely a dinuclear zinc‐carbene complex. The addition of methanol to a heteroleptic (NHC)‐ZnEt(Cl) pre‐catalyst generated the active species in situ. Low‐molecular‐weight PLLA (*M*_n_=18410 g mol^−1^) was degraded exclusively to oligomers (*M*_n_≈2000 g mol^−1^) and Me‐LA (28 %) after 24 h at room temperature. Ejfler and co‐workers^[145]^ explored the use of a homoleptic Zn^II^{ON}_2_ for the controlled transesterification of PLA into Me‐LA via an oligomeric precipitation strategy, using alcohol as an anti‐solvent. However, this process was limited to PLA of low molecular weight. Recently, Payne et al.^[146]^ reported a series of well‐defined mono‐ and dimeric Zn^II^‐Schiff base complexes for lactide polymerisation and PLA methanolysis. Schiff bases are traditionally easy to prepare and purify in high yield, and thus are ideal candidates for ligand scale‐up. Moreover, their functional versatility provides significant scope for catalyst fine‐tuning, and thus they lend themselves to the field of PLA recycling, which remains in its infancy.^[5]^ Interestingly, whilst dimers outperformed their monomeric counterparts in the polymerisation of *rac*‐LA, reduced activity was generally observed in the methanolysis of a PLA cup (*M*_n_ *=*45150 g mol^−1^). This was attributed to inferior catalyst stability, highlighting the importance of robust pre‐catalysts. Zn^II^{ON}_2_ (R=Cl, H) were identified as the outstanding candidates, achieving 100 % Me‐LA yield within 8 h at 80 °C in THF. It is anticipated the carbonyl is activated by the Lewis acidic Zn^II^‐centre, consistent with enhanced activity upon shifting from an electron‐donating (R=*t*Bu) to ‐withdrawing ligand backbone (R=Cl). McKeown et al.^[147]^ had previously reported a series of aminopiperidine‐based Zn^II^ and Mg^II^{ONN} complexes for lactide polymerisation. Extensive transesterification was observed during polymer purification, although ligand complexity precluded a complete degradation study. Recent work by Jones and co‐workers sought to simplify the ligand backbone with a particular focus on preserving activity. To this end, McKeown et al.^[148]^ developed a Zn^II^‐Schiff base complex bearing a simple ethylenediamine ligand, which exhibited high activity (TOF=114000 h^−1^) for lactide polymerisation. This was conducted under industrially relevant immortal conditions in the melt at 180 °C, demonstrating high catalyst tolerance, a desirable quality of a degradation catalyst. Román‐Ramírez et al.^[149]^ have subsequently performed an in‐depth kinetic study of PLA methanolysis using this Zn^II^‐complex. Experimental design identified temperature (40–130 °C) and catalyst loading (4–16 wt %) as the main variables influencing PLA degradation. Mass transfer limitations related to PLA particle size and stirring speed were considered negligible. Various PLA samples (*M*_n_=44350–71900 g mol^−1^) were degraded, achieving conversions up to 100 % Me‐LA within 1 h at 90 °C in THF. PLA consumption proceeded with a pseudo‐first‐order kinetic profile, whilst the production of Me‐LA was shown to proceed via a two‐step process through the intermediate formation of chain‐end groups (*E*_a_=39–65 kJ mol^−1^; Scheme 3). A subsequent study investigated the use of this Zn^II^‐complex in PLA methanolysis using various end‐of‐life sources (cup, toy and 3D printed material) between 70–110 °C.^[150]^ As expected, the largest deviations in Me‐LA selectivity and conversion were observed for the toy, which contained the highest number of additives. Recently, McKeown et al.^[151]^ demonstrated shifting to a propylenediamine analogue [R=N(H)Me] to have significant ramifications on activity. Indeed, rapid degradation of a PLA cup (*M*_n_=45150 g mol^−1^) was realised, obtaining 81 % Me‐LA yield within 30 min at 50 °C in THF. The corresponding ethylenediamine analogue exhibited significantly reduced activity (12 % Me‐LA in 6 h) under comparable conditions (4 wt % catalyst, 40 °C), highlighting the importance of structure–activity relationships.^[149]^ Substitution of the propylenediamine substituent (R=NMe_2_) resulted in reduced activity, although remained high, observing 84 % Me‐LA within 1 h. This system was scaled up to 12.5 g of PLA and found tolerant to the presence of PET. Scale up experiments have since used these Zn^II^‐complexes for the production of higher‐chain alkyl lactates including ethyl, propyl and butyl lactate.[^117^, ^152^] Removal of the amine group (R=H) resulted in a dramatic reduction in activity under identical conditions, implicating the amine group in the reaction. It is anticipated the Lewis acidic Zn^II^‐centre and amine group activate the incoming carbonyl group and alcohol respectively, analogous to the dual‐activation mechanism proposed for TBD (Figure 6). A recent kinetic study revealed these complexes to adopt unusual behaviour, noting curved Arrhenius plots and variable activation energies, whilst observing the formation of Me‐LA as low as −20 °C.^[153]^ Yang et al.^[154]^ recently reported Zn(HMDS)_2_ as a highly efficient catalyst for the transesterification of a variety of polyesters including PLA, poly(*β‐*butyrolactone) (PBL), poly(*δ*‐valerolactone) (PVL) and PCL. Promisingly, 99 % Me‐LA yield was achieved within 2 h at room temperature, although a high catalyst loading (1.0 mol %) and large excess of MeOH (24.7 equiv.) were used. The process was scaled up to 11.0 g of PLA (*M*_n_=49900 g mol^−1^) using 5 wt % catalyst, characterised firstly by the ROP of *rac‐*LA, followed by polymer purification and finally degradation with MeOH. Whilst promising, it is important to acknowledge PLA samples degraded were not commercially sourced and instead directly produced from *rac*‐LA. Consequently, the impact of additives and polymer processing on the amenability of the final PLA product to chemical recycling were not considered, and thus are not industrially representative. Zn(HMDS)_2_ also possesses a reasonably high market price (1 g, £123, Sigma Aldrich), limiting scalability.

Whilst significant developments have been made within the last 5 years, a number of challenges remain. Although consideration of mixed plastic waste streams on catalyst activity and selectivity is becoming increasingly assessed, it is imperative it becomes routine to overcome inevitable barriers to industrial application. Additionally, whilst the recovery and reuse of simple commercially available metal salts has been well established, it remains overlooked for discrete metal‐based systems. A possible solution to this is immobilisation on a support, although heterogeneous‐based systems for PLA alcoholysis remain scarce. The pursuit of more active and robust catalysts should assist in addressing this concern, ultimately targeting a system that operates in air under ambient conditions. Work has also primarily focused on zinc; however, concerns associated with its long‐term availability have created an appetite for metal diversification.^[155]^ Here, we argue prioritisation of cheap, earth‐abundant and environmentally benign metals (e. g., Mg, Fe, Ca) to ensure a sustainable future. For inspiration, the scientific community need not look further than the plentiful and diverse array of initiators reported for lactide polymerisation. A summary of the systems discussed in the preceding section is provided in Table 1.

**Table 1:** Summary of selected metal‐based and organocatalysts reported for PLA transesterification.^[a]^

Cataylst	MeOH/ester unit (*n*/*n*)	Cat. [mol %]	*T* [°C]	*t* [h]	PLA conv. [%]	*S*_Me‐LA_ [%]	*Y*_Me‐LA_ [%]	Ref.
ILs								
[Bmim][OAc]	5 : 1	2^[b]^	115	3	97	96	93	[123]
2[Bmim][OAc]‐Zn(OAc)_2_	5 : 1	1^[b]^	110	2	97	95	92	[124]
[H‐DBU][OAc]	5 : 1	5	100	5	100	91	91	[129]
[H‐DBU][Im]	5 : 1	10	70	1	100	87	87	[130]
Organocatalysts								
DMAP	23.2 : 1	5	180^[c]^	0.17	–	–	97	[128]
TMC	7 : 1	4^[b]^	50	1	100^[d]^	100^[d]^	100^[d]^	[131]
TBD	3 : 1	1	25	0.033	100	100	>95	[132]
Metal‐based								
Zn(OAc)_2_	5.3 : 1	1.4	65	15	90^[e]^	72	65	[134]
FeCl_3_	5 : 1	1	130	4	96	91	87	[135]
KF	23.1 : 1	1	180^[c]^	0.17	–	–	98	[136]
Sn(Oct)_2_	15.4 : 1	0.05	180^[c]^	0.017	–	–	33	[137]
bismuth subsalicylate	67.5 : 1	0.1	180^[c]^	0.017	–	–	23	[138]
Zn(OAc)_2_	67.5 : 1	0.1	180^[c]^	0.017	–	–	75	[139]
Hf(IV)‐salalen (R=Me)	25.5 : 1	1	25	24	>99^[f]^	75	75	[143]
(NHC)‐ZnEt(Cl) pre‐catalyst	0.5 : 1	0.5	25	24	89^[f]^	31	28	[144]
Zn^II^{ON}_2_ (R=Cl, H)	7 : 1	8^[b]^	80	8	100^[d]^	100^[d]^	100^[d]^	[146]
Zn^II^{ONN}_2_^Et^	7 : 1	8^[b]^	50	3	85^[d]^	45^[d]^	38^[d]^	[149]
Zn^II^{ONN}_2_^Pr^ (R=N(H)Me)	7 : 1	4^[b]^	50	0.5	100^[d]^	81^[d]^	81^[d]^	[151]
Zn^II^{ONN}_2_^Pr^ (R=NMe_2_)	7 : 1	4^[b]^	50	1	100^[d]^	84^[d]^	84^[d]^	[151]
Zn^II^{ONN}_2_^Pr^ (R=H)	7 : 1	4^[b]^	50	3	29^[d]^	17^[d]^	5^[d]^	[151]
Zn(HMDS)_2_	24.7 : 1	1	25	2	100	99	99	[154]

[a] *S*_Me‐LA_ and *Y*_Me‐LA_ refer to selectivity and yield of Me‐LA, respectively. Yields determined by ^1^H NMR analysis unless otherwise stated. [b] Cataylst loading reported as wt %. [c] Microwave irradiation, power: 850 W. [d] Values determined by ^1^H NMR (CDCl_3_) analysis following solvent removal in vacuo. [e] Depolymerisation by mass of PLA recovered. Initial waste stream contained 1 : 1 mixture of [PLA]/[PET]. [f] Depolymerisation based on Δ*M*_n_ [determined by gel permeation chromatography (GPC) in THF] before and after degradation.

### 3.4 Reductive depolymerisation

PLA degradation methods discussed thus far retain carbonyl functionality in the final product, either as a carboxylic acid (hydrolysis) or ester group (transesterification). Adjusting the reducing agent employed enables a diverse range of value‐added chemicals to be accessed directly from plastic waste.

**Figure 8:**
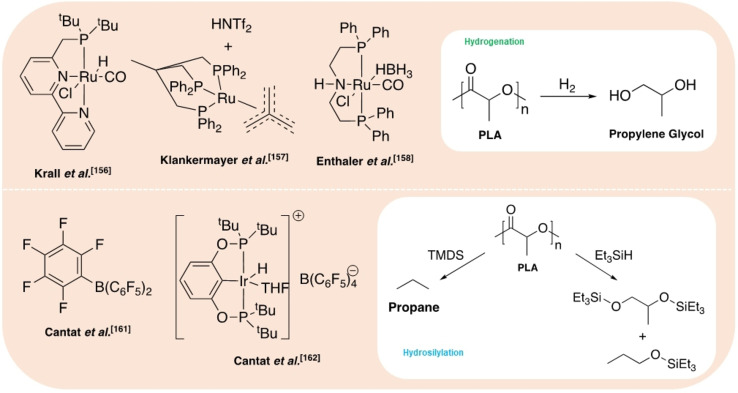
Selected metal‐based and organocatalysts reported for the hydrogenation (top) and hydrosilylation (bottom) of PLA.

Hydrogenation processes have been reported for the production of alcohols and alkanes (Figure 8). Krall et al.^[156]^ reported the use of a ruthenium(II)‐PNN pincer complex for the hydrogenation of various polyesters and polycarbonates. The active species is generated in situ by abstraction of the Cl ligand using KO*t*Bu. Employing a solvent mixture of THF and anisole, a PLA cup was successfully reduced to propylene glycol (PG). The active species is generated in situ by abstraction of the Cl (PG), achieving quantitative yield within 24 h at 160 °C and 54 bar (H_2_). Recently, Klankermayer and co‐workers^[157]^ investigated the use of a Ru^II^‐triphos complex for the recycling of polyesters and polycarbonates. Quantitative PG yield was achieved within 16 h at 140 °C and 100 bar (H_2_), employing either 1,4‐dioxane or PG as the reaction solvent, with bis(trifluoromethane)sulfonimide (HNTf_2_) as a co‐catalyst. Selective degradation of PLA in the presence of PET was demonstrated and scaled up to 11.4 g. Subsequently, Enthaler and co‐workers^[158]^ have applied a commercially available Ru‐MACHO‐BH complex to this process. This system was found to be tolerant to the presence of dyes and additives, observing PLA reduction to PG under significantly milder conditions with shorter reaction times (120–140 °C, 30–45 bar, <6 h). Mixed waste streams were also considered with a PLA and poly(propylene) mixture affording PG and MeOH. Shuklov et al.^[159]^ have exploited the use of a barium‐promoted copper chromite (Cu/Cr/Ba) heterogeneous catalyst at 150 bar (H_2_) for the reduction of PLA and lactide to PG. This process is characterised by a tandem reaction whereby Me‐LA is initially formed by methanolysis, which is subsequently reduced to PG via hydrogenation. A modest PG yield (50 %) was observed at 100 °C within 15 h, achieving 90 % *ee*. Reaction temperatures up to 150 °C were pursued, resulting in increased yield at the expense of severe product racemisation. The use of a high catalyst loading (133 wt %) is circumvented by facile recovery by centrifugation, a limiting feature of the homogeneous ruthenium‐based systems. Catalyst recyclability was demonstrated for lactide transformations. In principle, these processes make use of a waste feedstock to access green PG, which is traditionally produced from the petroleum‐based hydrogen peroxide propylene oxide (HPPO) process at a scale of approximately 1 million tonnes per year.^[159]^ Simple 1,2‐diols, such as PG, are high‐value speciality chemical intermediates used in a diverse range of applications, including the manufacture of biodegradable polyester fibres, unsaturated polyester resins and pharmaceuticals, to name but a few.^[160]^

Hydrosilylation strategies are also a possible route to higher‐value chemicals such as silyl ethers (Figure 8). Feghali and Cantat^[161]^ reported the first example of a metal‐free hydrosilylation process for a wide range of polyethers, polyesters and polycarbonates under ambient conditions, catalysed by B(C_6_F_5_)_3_. For PLA, the use of an air‐stable and inexpensive hydrosilane, namely 1,1,3,3‐tetramethyldisiloxane (TMDS), yielded propane in excellent yield (>99 %, 1 h) in CH_2_Cl_2_. Alternatively, substitution of TMDS for triethylsilane (Et_3_SiH) afforded silylated propylene glycol (Si‐PG), achieving 65 % yield within 16 h. Besides low energy intensity, a particular advantage of this recycling system is its tolerance to additives and mixed plastic waste. The same group has subsequently investigated the use of Brookhart's iridium(III) catalyst for PLA degradation, among other polymers.^[162]^ Using Et_3_SiH, a mixture of Si‐PG and propanol were formed at 65 °C in chlorobenzene after 60 h. Silylated propanol (*n*PrO−Si) was selectively formed at 90 °C in the presence of excess silane. PLA (3D printing material) was degraded despite the presence of additives, demonstrating high catalyst tolerance. As observed for B(C_6_F_5_)_3_, the use of TMDS afforded propane, although prolonged reaction times and higher temperatures were required (12 h, 110 °C), yielding a valuable silicon polymer as a by‐product, namely polydimethoxysilane (PDMS).

Whilst such methods demonstrate the versatile products accessible from plastic waste, the use of scarce and expensive rare‐earth metals, in combination with often complex ligands, is clearly undesirable. Moreover, such processes typically rely on harsh reaction conditions/toxic reagents, providing significant scope for optimisation. To this end, Nunes et al.^[163]^ recently reported a cheap, reusable and environmentally benign dioxomolybdenum complex {MoO_2_Cl_2_(H_2_O)_2_} for the reductive depolymerisation of PLA into propane using various silanes on a gram scale. This method further demonstrates the potential to access products traditionally derived from depleting fossil fuel resources, whilst simultaneously making use of polymethylhydroxysilane (PMHS), a by‐product of the silicone industry. PLA from various end‐of‐life sources (cup and 3D printing material) were degraded, requiring prolonged reaction (20–40 h) at 110 °C in toluene, whilst PG was implicated as a potential reaction intermediate. A summary of the systems discussed in the preceding section is provided in Table 2.

**Table 2:** Summary of selected metal‐based and organocatalysts reported for the hydrogenation and hydrosilylation of PLA.

Cataylst	Cat. [mol %]	Solvent	*T* [°C]	H_2_ [bar]	*t* [h]	PLA conv. [%]	Product(s)	Yield^[a]^ [%]	Ref.
Hydrogenation									
Ruthenium(II)‐PNN pincer	2^[b]^	anisole/THF	160	54.4	24	100	PG	>99	[156]
Ru^II^‐triphos complex	1^[c]^	1,4‐dioxane or PG	140	100	16	100	PG	>99	[157]
Ru‐MACHO‐BH complex	0.5	THF	140	45	3	100	PG	>99	[158]
(Cu/Cr/Ba) heterogeneous catalyst	133^[d]^	MeOH	100	150	15	–	PG	50^[e]^	[159]
Hydrosilylation				Silane (equiv.)					
B(C_6_F_5_)_3_	2	CH_2_Cl_2_	25	TMDS (2.0)	1	100	propane	>99^[f]^	[161]
	5	CH_2_Cl_2_	25	Et_3_SiH (3.3)	16	–	Si‐PG	65	[161]
Brookhart's iridium(III) catalyst	0.5	chlorobenzene	65	Et_3_SiH (3.0)	60	100	Si‐PG/*n*PrO‐Si	64 : 31	[162]
	1	chlorobenzene	90	Et_3_SiH (excess)	60	100	*n*PrO‐Si	92	[162]
MoO_2_Cl_2_(H_2_O)_2_	2	toluene	110	PMHS (2.0)	40	–	propane	95	[163]
	1	toluene	100	PhSiH_3_ (1.5)	20	100	propane	100	[163]

[a] Yields determined by ^1^H NMR analysis unless otherwise stated. [b] KO*t*Bu employed as a co‐catalyst in a loading ratio of 50 : 1 : 2 {[ester repeat unit]/[catalyst precursor]/[KO^t^Bu]}. [c] HNTf_2_ employed as a co‐catalyst in a loading ratio of 100 : 1 : 1 {[ester repeat unit]/[catalyst precursor]/[HNTf_2_]}. [d] Catalyst loading reported as wt %. [e] PG yield based on GC. [f] Propane yield based on GC‐MS analysis.

### 3.5 Other products

In the absence of exogeneous reagents, thermal degradation methods have been widely reported for the chemical recycling of PLA. High reaction temperatures are required, often affording lactide amongst other products, owing to competing side reactions and potential racemisation/epimerisation. We direct the interested reader to an excellent Review by McKeown and Jones^[89]^ that provides a succinct overview of the thermal degradation mechanisms discussed herein. Pioneering work by McNeill and Leiper investigated the thermal degradation of PLA between 250–450 °C under programmed heating conditions (10 °C min^−1^).^[164]^ PLA degradation was found to proceed in one step and product distribution was temperature dependent, confirmed by isothermal studies.^[165]^ CO_2_ was observed as the major product, with lactide and cyclic oligomers also present. Acetaldehyde formation via *cis*‐elimination was observed at 230 °C with higher temperatures favouring the formation of CO_2_. Short chain alkenes such as ethylene, propylene and methyl ketene were also observed at higher temperatures. Thermal degradation proceeded via a back‐biting mechanism, confirmed by acetylation of the chain ends enhancing polymer thermal stability by approximately 30 °C. Subsequent work in the field has investigated the addition of simple metal salts on thermal degradation characteristics, with a particular focus on polymer processability at end‐of‐life.[^166^, ^167^, ^168^, ^169^, ^170^, ^171^, ^172^, ^173^, ^174^, ^175^, ^176^, ^177^] Industrially, PLA production relies on a Sn(Oct)_2_ catalyst and thus residual Sn^II^ species in the final polymer are common. Trace metal residues often adversely impact polymer thermal stability, reducing the onset degradation temperature. Nishida et al.[^167^, ^176^] have previously shown the selective formation of l *‐*LA from PLLA via an intramolecular unzipping mechanism mediated by tin, contrasting random intermolecular transesterification. Sn^II^ carboxylate end groups were found to drastically reduce the activation energy (from 175 to 85 kJ mol^−1^, depending on tin concentration), enabling onset weight loss as low as 150 °C. Poorer depolymerisation control was noted in the absence of Sn^II^, favouring the formation of oligomers and *meso*‐LA. Calcium and magnesium oxides have also been shown to operate via an unzipping mechanism, observing a comparable activation energy trend relative to tin.[^171^, ^173^, ^175^] Product racemisation was found to be both temperature and metal dependent. CaO was found to selectively form l *‐*LA at high temperatures (<300 °C); however, extensive *meso*‐LA formation was noted below 250 °C. Conversely, racemisation with MgO was less prevalent and high selectivity towards l *‐*LA was retained below 270 °C. Trace residual organocatalyst (DBU) and Zn^II^, Fe^III^ and Al^III^ cations have also been explored for PLA pyrolysis.[^169^, ^177^, ^178^]

Pyrolysis potentially represents a route of least resistance to tackling plastic waste due to existing industrial precedent. However, such processes are energy intensive and offer limited value return for PLA, often returning the cyclic monomer, lactide. Beyond pyrolysis, Enthaler and co‐workers[^139^, ^179^] have demonstrated lactide recapture is possible via microwave irradiation in the presence of zinc‐based salts, achieving TOFs up to approximately 260 h^−1^ between 200–210 °C. However, where possible, it is imperative to pursue upcycling for emerging products to promote market penetration through economic incentives to industry. Recent work by Slater et al.^[180]^ reported the synthesis of high‐value lactate‐containing metal–organic frameworks (MOFs) from waste PLA, further highlighting the potentially vibrant product portfolio attainable from waste PLA. Alkyl lactyllactates, the dimeric precursor to lactate esters, have also attracted interest owing to properties akin to their monomer.^[5]^ Group I,[^119^, ^141^] group II[^119^, ^120^, ^121^, ^141^] and Al^III[141,181,182]^ have been reported, although primarily limited to the alcoholysis of lactide, with the exception of work by Sobota and co‐workers.^[141]^ We identify this as an emerging area of opportunity, particularly with regards to translating such catalysts to PLA. Indeed, catalysts that exhibit modest activity are perhaps desirable where controlled and selective partial depolymerisation is required. A tailored approach to catalyst development will undoubtedly provide use to systems that might otherwise be overlooked.

## 4 Chemical Recycling of Poly(ethylene terephthalate)

### 4.1 Poly(ethylene terephthalate)

**Figure 9:**
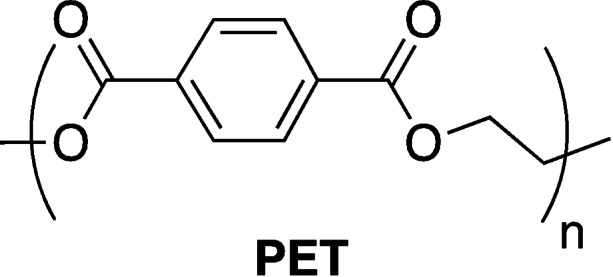
Polymeric structure of PET.

Whilst the development of recycling technologies in tandem with emerging bioplastics is central to the industry's transition, there is also a pressing need to address waste concerns associated with the current product portfolio. Indeed, bio‐based plastics accounted for just 1 % of all processed plastics in 2019.^[63]^ PET (Figure 9) is a commercially important polyester, which exhibits high mechanical strength, good barrier properties and high optical clarity.[^42^, ^183^] Consequently, PET has been widely exploited in the packaging industry, which consumed 38 % of plastics produced globally in 2015, with PET accounting for 22.6 % of plastic use in the sector.^[11]^ PET has also found use in the construction, transport and textiles industry.^[183]^ Industrially, PET is manufactured via a four‐step process. Firstly, bis(2‐hydroxyethyl) terephthalate (BHET) is produced from the esterification of ethylene glycol (EG) with terephthalic acid (TA). Transesterification of EG with dimethyl terephthalate (DMT) was widely used up until the 1960s, although slower reaction rates and high corrosivity rendered it obsolete. The second and third stage are characterised by the pre‐polymerisation of BHET and subsequent melt condensation to form low‐*M*_n_ PET (suitable for fibres), respectively. Finally, solid‐state polymerisation is used to access PET of high *M*_n_ suitable for drinks bottles.[^42^, ^184^] For PET synthesis, antimony‐based catalysts, such as Sb_2_O_3_ and Sb(OAc)_3_, are generally considered the most effective and thus are routinely used.^[185]^

Traditionally, EG and TA are sourced from petroleum‐based feedstocks, although the synthesis of bio‐based PET is possible. Presently, Bio‐PET in circulation is only 30 % bio‐based (Bio‐PET30), corresponding to renewably sourced EG from biomass, and is currently marketed by several well‐known brands such as Coca‐Cola and Pepsi.[^42^, ^186^, ^187^] Whilst 100 % bio‐based PET remains a long‐term ambition of the industry, technical constraints associated with renewable TA production have limited commercialisation. Promisingly, Bio‐PET is compatible with existing processing and recycling equipment, although it remains non‐biodegradable. This serves to highlight that bio‐based polymers are not inherently biodegradable and that irresponsibly handled PET waste is a major source of plastic pollution.

The mechanical recycling of PET is well established but is limited by eventual material downcycling, with ductility decreasing from 310 to 2.9 % after just three cycles. This necessitates recycled PET be repurposed into lower‐value products, such as fibres (72 %) in carpeting, which can no longer be recycled.[^42^, ^188^] Moreover, PET waste streams are easily contaminated by PVC and PLA, rendering the recycled product of low‐grade quality, which can no longer be mechanically recycled.^[42]^ However, the commercial viability of mechanical recycling relies on a high (≥$75 per barrel) and stable oil price. Below $65 per barrel, the economics become challenging, which inhibits recycling efforts as noted in 2015.^[46]^

A possible solution to this is chemical recycling. Beyond long‐term material value retention, the possibility of accessing higher‐value products offers a potential route to decoupling PET recycling from a volatile oil market. Addressing this clear industry appetite is paralleled by the opportunity to enact timely and meaningful action. Relative to PLA, there is an exhaustive body of literature concerning the chemical recycling of PET, and we highlight a number of excellent Reviews.[^44^, ^184^, ^189^, ^190^, ^191^, ^192^, ^193^] We do not intend to reproduce such work but instead provide a brief overview of traditional methods with a particular focus on upcycling and recent developments in catalysis.

### 4.2 Hydrolysis to terephthalic acid

Traditionally, PET hydrolysis requires high‐temperature (200–250 °C) and ‐pressure regimes (1.4–2 MPa) under either acid, basic or neutral conditions to afford TA and EG.^[189]^ Acid hydrolysis is typically facilitated by concentrated H_2_SO_4_ (minimum 87 wt %), although other inorganic acids such as HNO_3_ and H_3_PO_4_ have been reported.[^44^, ^189^, ^194^, ^195^] A major limitation of this method is the large quantities of inorganic and aqueous waste produced, coupled with high system corrosivity. Alkaline hydrolysis typically relies on a solution of NaOH or KOH of a concentration between 4–20 wt % to afford EG and the corresponding disodium or dipotassium terephthalate salt.[^189^, ^192^, ^196^, ^197^] EG can be recovered via distillation, whilst pure TA can be isolated by neutralisation of the reaction mixture with a strong inorganic acid (e. g., H_2_SO_4_). This method can tolerate highly contaminated post‐consumer PET such as metallised PET film.^[189]^ Neutral hydrolysis involves the use of water or steam in the presence of a transesterification catalyst, typically an alkali metal acetate.[^44^, ^192^, ^198^, ^199^] This method remedies concerns associated with equipment corrosion and waste disposal prevalent in acid and alkali‐based methods. However, the process is limited by low TA purity, necessitating further purification at the expense of increased process cost and complexity.^[189]^ Consequently, hydrolysis is not widely used in industry for the production of food‐grade recycled PET. Comparatively, hydrolysis is a slow process due to water being a poor nucleophile. Enzymatic‐based processes (PETase) have also been reported. Recently, Tournier et al.^[200]^ reported the fastest PETase to date, capable of achieving a minimum of 90 % conversion to monomers within 10 h, equating to a productivity of 16.7 g L^−1^ h^−1^. This represents a remarkable improvement relative to previously reported systems, which exhibited limited productivity, highlighting the rapidly progressing field of biocatalysis as a possibly feasible bioremediation strategy in the future.[^201^, ^202^, ^203^, ^204^]

### 4.3 Methanolysis to dimethyl terephthalate

As noted for hydrolysis, PET methanolysis relies on high temperatures (18–280 °C) and pressures (2–4 MPa) to afford DMT and EG, which can be used as raw starting materials for polymer production.[^44^, ^205^, ^206^] Zinc acetate is most commonly employed as a transesterification catalyst; however, magnesium acetate, cobalt acetate, lead dioxide and aryl sulfonic acid salts have also been reported.^[189]^ Recently, McKeown et al.^[131]^ reported the first example of organocatalysed PET methanolysis using TMC (Figure 6). Promisingly, DMT could be isolated in good yield (72 %) at temperatures as low as 100 °C under ambient pressure, although prolonged reaction times (16 h) were required. PET depolymerisation in supercritical methanol has also been reported, generally observing enhanced reaction rates due to higher density and kinetic energy in the supercritical state.[^189^, ^207^, ^208^, ^209^, ^210^] Due to the propensity of DMT to undergo transesterification, catalyst deactivation is required following reaction termination. Whilst the process is reasonably tolerant to contaminants, water perturbs the process, resulting in catalyst deactivation and the formation of various azeotropes. A limiting feature of this process is the resulting complex product feed, comprising glycols, alcohols and phthalate derivatives, which renders DMT separation both costly and time consuming.[^44^, ^189^] Presently, the cost of methanolysis‐derived DMT is approximately double that of virgin DMT, and thus is unable to compete with cheap petroleum feedstocks. Moreover, market penetration is limited by manufacturers favouring TA as a feedstock for PET production due to greater process performance. Whilst DMT can be hydrolysed to TA, this incurs considerable additional cost to the process. Consequently, the use of methanolysis‐derived DMT as a feedstock in the future relies on technological innovation and a high oil price, or indeed a shift from petroleum entirely.

### 4.4 Ammonolysis to terephthalamide

PET ammonolysis typically employs liquor ammonia between 70–180 °C under pressure (2 MPa) in either the presence or absence of catalyst, typically zinc acetate.[^189^, ^211^] The main degradation products are 1,4‐benzene dicarboxamide, otherwise known as terephthalamide (TPA), and EG. TPA serves as an intermediate to terephthalonitrile, which can be subsequently reduced via hydrogenation into either *p*‐xylenediamine or 1,4‐bis(aminomethyl)cyclohexane. Low‐pressure ammonolysis is also possible using ammonia in an EG environment, catalysed by zinc acetate (0.05 wt %). TPA was recovered in 87 % yield at 70 °C using a PET/NH_3_ ratio of 1 : 6.^[189]^ Whilst not directly amenable to polymer reprocessing, TPA and its derivatives represent potentially useful building blocks for the production of both saturated and unsaturated terephthalamides, which are discussed in further detail herein. Unsurprisingly, PET ammonolysis has received little interest in the literature, likely due to limited substrate scope and commercial interest.

### 4.5 Aminolysis to diamines of terephthalic acid

Presently, there are no known examples of PET aminolysis use at a commercial scale. However, partial aminolysis is exploited for enhancing PET properties (e. g., fibre colouration) in the manufacture of fibres with defined processing properties.[^189^, ^191^] Aminolytic chain cleavage of PET affords diamines of TA and EG and is thermodynamically more favourable than alcoholysis owing to enhanced nucleophilicity. Consequently, aminolysis is typically conducted under milder reaction conditions (20–100 °C) in both the presence or absence of catalyst. Commonly used aqueous solutions of primary amine include methylamine, ethylamine, ethanolamine and anhydrous *n‐*butylamine.[^44^, ^189^]

Fukushima et al.^[212]^ have reported the organocatalysed aminolysis of PET waste mediated by TBD, employing a diverse range of aliphatic, allylic and aromatic amines. Typical reaction conditions afforded 63–89 % yield within 1–2 h at 110–120 °C. The resulting crystalline terephthalamides exhibited attractive thermal (m.p. up to 301 °C) and mechanical properties with potential uses as additives, modifiers and building blocks for high‐performance materials. The origin of these desirable properties was attributed to amide hydrogen bonding and structural rigidity of the monomer. Further computational study concluded the bifunctionality of TBD plays a crucial role in aminolysis, in particular activation of the carbonyl, differentiating TBD from other organic bases such as DBU. Such behaviour had previously been discussed for PLA degradation mediated by TBD (Figure 6). Deep eutectic solvents (DES) have also been reported as highly efficient organocatalysts for PET aminolysis. Musale and Shukla^[213]^ employed choline chloride ⋅ 2 ZnCl_2_ (5 wt %) in the production of *N* ^1^,*N* ^1^,*N* ^4^,*N* ^4^‐tetrakis‐(2‐hydroxyethyl)‐terephthalamide (THETA) and TA, and bis(2‐hydroxyethylene) terephthalamide (BHETA), achieving 82, 83 and 95 % yield within 30 min under reflux (PET/amine=1 : 6).

Metal‐mediated examples of PET aminolysis include sodium acetate, potassium sulfate and dibutyl tin oxide for the production of BHETA using excess ethanolamine (EA).[^214^, ^215^, ^216^] BHETA has possible uses as an environmentally benign corrosion inhibitor for the protection of steel structures. This is highly desirable as powerful corrosion inhibitors tend to be toxic and carcinogenic.^[216]^ Microwave‐assisted methods for PET aminolysis have also been reported. Cheap and non‐toxic simple metal salts, such as sodium acetate, sodium bicarbonate and sodium/potassium sulfate are frequently used, achieving excellent product yield (>85 %) within minutes.[^217^, ^218^, ^219^] Heterogeneous and recyclable β‐zeolite acid catalyst and montmorillonite KSF clay catalyst have also been reported, affording BHETA in good yield (85–88 %).^[220]^ Here, the resulting product was found to undergo a cyclisation reaction under reflux mediated by polyphosphoric acid to produce 2,2’‐(1,4‐phenylene)‐bis(2‐oxazoline) (PBO), a possible chain extender/coupling agent or cross‐linker.

An excellent Review by George and Kurian^[44]^ highlights the possible applications of PET aminolysed products, which include antibacterial drugs,^[221]^ adhesion promoters^[222]^ and polyol components for rigid polyurethane foams.^[223]^ Despite the diverse chemistry and breadth of applications of aminolysis‐derived products, the area remains vastly underexplored. Additionally, to the best of our knowledge, there are no known examples of homogeneous PET aminolysis mediated by discrete metal‐based complexes. Thus, there is clear scope for further catalyst optimisation. Surprisingly, no examples of PET thiolysis or phosphorolysis have been reported to date. We identify these as potential avenues for accessing vibrant and diverse products of untapped potential. The impact of mixed plastic waste on the activity and recyclability of these catalysts remains unaddressed.

### 4.6 Glycolysis to bis(2‐hydroxyethyl) terephthalate

PET glycolysis is a well‐established commercial process operated by a number of leading global companies such as DuPont, Shell and Eastman Kodak.^[44]^ Indeed, the first patents detailing PET glycolysis were filed over 50 years ago, rendering it the oldest recycling method for PET.[^224^, ^225^, ^226^, ^227^] Consequently, glycolysis is the most widely used chemical recycling method for PET, characterised by cleavage of the ester bond via insertion of a glycol, most commonly EG, to produce BHET.^[44]^ Higher‐chain alcohols such as PG and 1,4‐butanediol (BD) have also been reported.[^228^, ^229^] Typically, high temperatures (180–240 °C) and prolonged reaction times (0.5–8 h) in the presence of a transesterification catalyst, often a metal acetate, are required to achieve appreciable conversion. Whilst numerous metal acetate catalysts have been reported in the literature, zinc acetate is considered the benchmark.[^42^, ^44^, ^189^] Additionally, a large excess of EG (EG/PET ≥5 : 1) is used to mediate the formation of higher chain oligomers, thus favouring the formation of BHET.^[42]^ The method lends itself to the recovery of post‐industrial PET waste where the incoming feed is of known origin and high quality.^[44]^

Organocatalysts have been widely reported as efficient catalysts for PET glycolysis. Wang et al.^[230]^ employed urea (10 wt %) as a cheap and reusable catalyst, achieving 100 % PET depolymerisation and approximately 80 % BHET yield under optimal reaction conditions [3 h, 180 °C, *m*(PET)/*m*(EG)=1 : 4]. DFT and complementary experimental study revealed hydrogen bond formation between EG and urea played a crucial role in the enhanced reaction rate. In 2011, Fukushima et al.^[231]^ explored the use of a commercially available guanidine for PET glycolysis, namely TBD (1.0 mol %). After 3.5 h at 190 °C, BHET was isolated in 78 % yield following recrystallisation to remove residual impurities (e. g., oligomers and additives). The observed activity was comparable to that reported for commonly used metal acetates/alkoxide catalysts. The excess of unreacted EG and TBD catalyst could be recycled more than 5 times. Further computational study confirmed TBD and EG activate PET through hydrogen bond formation, consistent with previous studies.[^132^, ^212^, ^230^] Prior work in this group reported a highly efficient N‐heterocyclic (NHC) carbene catalyst derived from a commercially available imidazolium IL.^[232]^ NHC catalysis enabled PET glycolysis to be conducted under reflux in anhydrous tetrahydrofuran to afford BHET, noting significantly milder reaction conditions and a shortened reaction time of 1 h. The commercial potential of NHC catalysis for PET depolymerisation is reflected in a patent filed in 2006.^[233]^ Indeed, nucleophilic NHCs have previously been exploited for the production of PET via the transesterification of DMT with EG.^[234]^ Beyond TBD and NHCs, a comprehensive study by Fukushima et al.^[235]^ investigated other nitrogen‐containing bases for PET glycolysis, including DMAP, DBU and 1,5‐diazabicyclo[4.3.0]non‐5‐ene (DBN) to name but a few. Recently, TBD and DBU have been explored as catalysts for transesterification and amidation reactions using EG, EA and ethylenediamine, employing methylbenzoate as a model system for PET.^[236]^ Whilst traditional organocatalysts generally exhibit high activity for PET glycolysis, they remain limited by activity loss incurred over repeated use due to oxodegredative reactions or competing side reactions.^[22]^ The most recent advancements in this area concerns the development of amidine and guanidine‐type eutectic salts, which exhibit superior stability and efficiency.[^235^, ^237^] Jehanno et al.^[237]^ reported the first example of an industrially relevant organocatalyst, namely a TBD/methanesulfonic acid complex (TBD/MSA, 1 : 1). This protic ionic salt combined the high catalytic activity of the free base with superb thermal stability (>400 °C), achieving 91 % BHET yield within 2 h at 180 °C. Moreover, the catalyst could be recycled at least 5 times.

**Figure 10:**
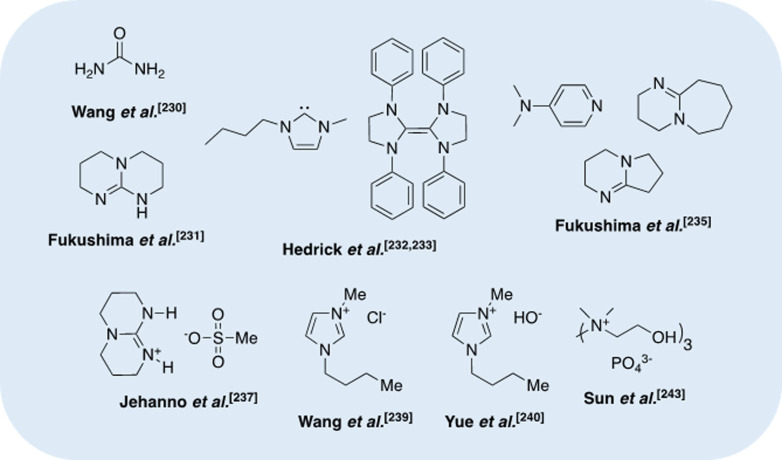
Selected organocatalysts reported for PET glycolysis.

Unsurprisingly, ILs have also seen extensive use as catalysts for PET glycolysis.[^230^, ^238^, ^239^, ^240^, ^241^, ^242^, ^243^] Beyond benefits noted for PLA, the reaction products are easily separated from the IL by addition of water followed by filtration, enabling facile catalyst recovery and reuse. This is a limiting feature of glycolysis catalysed by traditional compounds such as metal acetates. Wang et al.^[238]^ investigated the use of acidic, basic and neutral ILs in the glycolysis of waste PET using EG. Acidic ILs exhibited poor stability above 180 °C, whilst basic ILs were limited by a complex and high production cost. Neutral ILs were preferred based on cost and performance. [Bmim][Cl] was selected as the ideal catalyst due to its high stability, despite exhibiting inferior performance to [Bmim][Br], which achieved 99 % PET depolymerisation within 8 h at 180 °C.^[239]^ No reaction occurred between PET and the IL, and depolymerisation proceeded to be first‐order using [Bmim][Cl] (*E*_a_=232.79 kJ mol^−1^). Yue et al.^[240]^ further explored basic ILs as degradation catalysts, identifying [Bmim][OH] as the outstanding candidate. Under the optimal conditions, 71 % BHET yield was obtained within 2 h at 190 °C, although a relatively high catalyst loading (5 wt %) is noted. Recently, ILs embedded with first‐row transition metals (e. g., Fe, Co and Zn) have been reported.[^241^, ^242^] Generally, enhanced activity is observed relative to traditional ILs, attributed to the presence of a Lewis acidic metal centre facilitating enhanced nucleophilic attack. For example, Wang et al.^[241]^ noted [Bmim]_2_[CoCl_4_] achieved 81 % BHET yield within 1.5 h at 175 °C and could be recycled up to 6 times. Enhanced thermal stability was also noted, promoting industrial relevance. A common drawback of ILs is their high cost. However, Sun et al.^[243]^ recently reported a low cost ($1.2 kg^−1^) and biocompatible IL, cholinium phosphate ([Ch]_3_[PO_4_]) for the glycolytic degradation of PET. Under metal‐free conditions, 61 % BHET yield was achieved within 4 h at 180 °C. A low catalyst cost is paramount if ILs are to be considered industrially viable in the future given the high catalyst loadings commonly reported (20–25 wt %).[^238^, ^239^, ^241^, ^242^, ^243^] Recent work has focused on DESs as a cheaper, less toxic and often biodegradable alternative to ILs.[^244^, ^245^] Recently, Wang et al.^[244]^ reported the use of 4(urea) ⋅ (ZnCl_2_) (5 wt %) for PET glycolysis, obtaining 83 % BHET yield within 30 min at 170 °C. This reaction time is equivalent to that taken by a supercritical method under 15.3 MPa at 450 °C, highlighting the importance of catalysis to optimising process efficiency.^[246]^ Zhou et al.^[245]^ further extended the scope of DESs for the production of dioctyl terephthalate (DOTP), a green and non‐toxic plasticizer, from PET waste using 2‐ethyl‐1‐hexanol. A summary of the organocatalysts discussed for PET glycolysis is provided in Figure 10.

PET glycolysis mediated by metal‐based catalysts has also been reported. Pingale et al.^[247]^ investigated the use of various metal chlorides (e. g., Zn, Li, Mg and Fe) for the catalytic degradation of waste PET bottle. Zinc chloride (0.5 wt %) was found to be most active, achieving 73 % BHET yield within 8 h under reflux (197 °C, *n*(PET)/*n*(EG)=1 : 10). A reactivity series of Zn>Pr/Nd>Mg>Li>Fe was proposed, although the optimal PET/EG molar ratio varied with metal type. Whilst such salts are cheap and readily available, they remain limited by slow reaction rates, harsh reaction conditions and difficulties associated with catalyst recovery. Consequently, subsequent research has sought to address such concerns. Pingale and Shukla^[248]^ explored the use of environmentally friendly catalysts such as sodium carbonate and sodium bicarbonate. The latter afforded 65 % BHET yield, competitive with zinc acetate. More importantly, microwave‐assisted depolymerisation (800 W) enabled the reaction time to be reduced from 8 h to 35 min relative to conventional electric heating under identical conditions (5 wt % catalyst, PET/EG=1 : 6). In a subsequent study, Loṕez‐Fonseca et al.^[249]^[JP1]  extensively investigated the use of simple and eco‐friendly metal salts, such as sodium and potassium sulfate, for PET glycolysis at a reasonably large scale (30 g). Comparably high BHET yield (≈70 %) was obtained within 1 h at 196 °C using zinc acetate and sodium carbonate (1 mol %) in the presence of a large excess of EG. Zhu et al.^[250]^ have previously reported a series of recyclable solid acid catalysts including sulfated oxides of zinc (SO_4_^2−^/ZnO), titanium (SO_4_^2−^/TiO_2_) and their binary oxide (SO_4_^2−^/ZnO‐TiO_2_). SO_4_^2−^/ZnO‐TiO_2_‐200–300 °C (note 200–300 °C refers to calcination temperature range) exhibited the highest catalytic activity with a PET conversion and BHET selectivity of 100 and 72 %, respectively, after 3 h at 180 °C under atmospheric pressure. Catalytic activity was attributed to a high surface area and predominance of Lewis acid sites. Whilst the catalyst could be reused up to four times, potential pollution concerns coupled with their corrosive nature limits scalability. Porous structures such as zeolites (e. g., β‐zeolite and γ‐zeolite) have been investigated as environmentally friendly alternatives that retain a high surface area for PET glycolysis.^[251]^

Recently, nanoparticles have received increasing attention as heterogeneous transesterification catalysts for PET glycolysis owing to their facile preparation, high surface area and recyclability. Bartolome et al.^[252]^ reported the use of superparamagnetic γ‐Fe_2_O_3_ nanoparticles (average size: 10.5±1.4 nm, surface area: 147 m^2^ g^−1^) for PET glycolysis. The reaction proceeded at 300 °C and 1.1 MPa, achieving >90 % BHET yield within 1 h using an exceptionally low catalyst loading (0.05 wt %).

High catalytic activity was attributed to the γ‐Fe_2_O_3_ nanoparticles ability to facilitate glycolysis via redox reactions, high area surface promoting more active sites, thermal stability and good crystallinity. Promisingly, the γ‐Fe_2_O_3_ nanoparticles could be easily separated by magnetic decantation post reaction and were reused 10 times. Metal‐oxide doped silica nanoparticles (Mn_3_O_4_/SNPs) have also been investigated as recoverable transesterification catalysts for PET degradation.[^253^, ^254^] Metal oxides of zinc, manganese and cerium were deposited on silica nanospheres of various diameters (60–750 nm) using ultrasonic irradiation. Manganese oxide‐doped silica nanoparticles (1 wt %) afforded the highest BHET yield (>90 %), observing equilibration within 80 min at 300 °C and 1.1 MPa. Smaller nanosphere supports promoted superior catalyst distribution, likely due to a higher surface area/volume ratio, resulting in enhanced activity. Imran et al.^[255]^ have reported mesoporous mixed‐metal oxide spinels of manganese, cobalt and zinc as novel catalysts for PET glycolysis. ZnMn_2_O_4_ was found to be most active, yielding 92 % BHET within 60 min at 260 °C and 5 atm. It was found that the cation pair, positioning within the crystal structure and spinel geometry influenced catalytic efficiency. Despite ease of catalyst recovery and reuse, such systems remain limited by their high energy intensity (260–300 °C, 1–5 atm). Recent developments include the utilisation of Fe_3_O_4_‐boosted multiwalled carbon nanotubes (MWCNT) and ultrasmall cobalt nanoparticles for PET glycolysis under milder reaction conditions.[^256^, ^257^] Indeed, for the former, 100 % BHET yield was obtained within 2 h at 190 °C, whilst the catalyst could be reused in at least 8 sequential runs.^[256]^ Promisingly, the latter reported a water‐free BHET precipitation method that enabled direct reuse of the remaining EG solution, thus simplifying product separation.^[257]^

**Figure 11:**
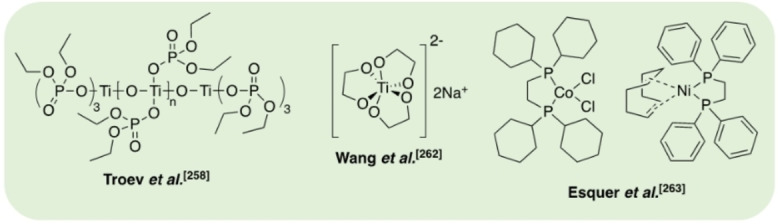
Selected examples of discrete metal‐based catalysts reported for PET glycolysis.

Despite the extensive metal‐based systems previously discussed, examples of PET glycolysis mediated by discrete metal‐based complexes remain rare (Figure 11). Most notably, Troev et al.^[258]^ reported a Ti^IV^‐phosphate catalyst (0.3 wt %) for the glycolysis of PET fibres, achieving 98 % conversion to BHET within 150 min between 190–200 °C, outperforming Zn(OAc)_2_ (PET/EG=1 : 3). No noticeable change in depolymerisation activity relative to Zn(OAc)_2_ was observed upon shifting to bottle‐grade PET, although greater optical clarity in isolated BHET was noted. The design premise of this catalyst was to combine the high activity of traditional titanium alkoxides with a thermal stabiliser (e. g., trialkyl phosphate) to circumvent undesirable yellowing in the product arising from competing side degradation reaction. Indeed, simple titanium alkoxides [e. g., titanium‐butoxide (TBT)] have been reported as highly active catalysts for PET degradation.[^259^, ^260^, ^261^] Consequently, this work represents an excellent example of addressing industry challenges through judicious catalyst design. Wang et al.^[262]^ reported sodium titanium tris(glycolate) [Ti(OCH_2_CH_2_O)_3_Na_2_] as a catalyst for PET recycling via glycolysis. This catalyst offered significantly higher activity than sodium carbonate or tetrabutyl titanate, ascertaining 85 % BHET yield within 3 h at 190 °C (1 mol % catalyst loading, PET/EG=1 : 12). Ti(OCH_2_CH_2_O)_3_Na_2_ was found to be more tolerant to lower catalyst loadings relative to zinc acetate, although it was generally outperformed. Promisingly, Ti(OCH_2_CH_2_O)_3_Na_2_ could also be used to re‐polymerise BHET to form recycled PET (rPET), culminating in a completely circular recycling strategy. Recently, Esquer and García^[263]^ reported the use of commercially available phosphine ligands [e. g., 1,2‐bis(dicyclohexylphosphino)ethane (dcype) and 1,2‐bis(diphenylphosphino)ethane (dppe)] in combination with cheap and air‐stable metal pre‐catalysts, for example CoCl_2_. Typical reaction conditions used 1.5 mol % catalyst and a large excess of EG, obtaining poor to good BHET yield (10–75 %) within 3 h at 190 °C. Monodentate ligands typically afforded lower BHET yields relative to bidentate ligands, which varied with the metal precursor, with Co‐based systems generally appearing more active. Whilst this system is limited by poorer activity relative to previous systems, the use of air stable reagents is desirable. Polymer scope was expanded to polyurethane for the production of polyols. In all instances, no attempt to recover the homogeneous catalyst were made. Thus, challenges associated with catalyst recovery and colour in the final product remain. Whilst heterogenization is a possible solution, such catalysts do not often maintain high BHET selectivity and typically require higher temperatures than other catalysts discussed.

**Figure 12:**
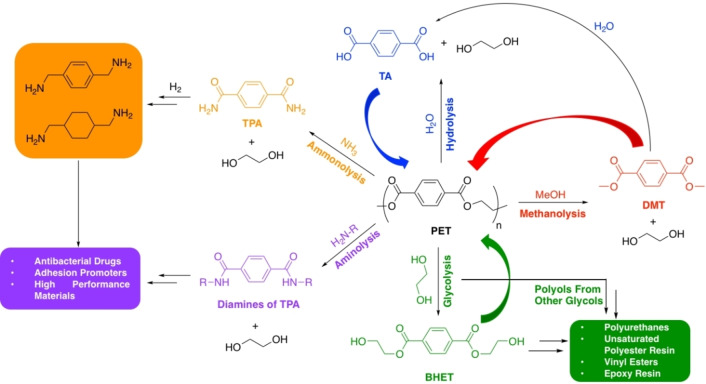
Summary of chemical recycling options for PET with example applications of value‐added products. Note: green, red and blue arrows denote recycled products directly amenable to polymer reprocessing.

Example applications of PET glycolyzed products include the production of unsaturated polyester resins,[^228^, ^264^, ^265^] polyurethanes,[^266^, ^267^] epoxy resins,^[268]^ vinyl esters,^[269]^ polymer concretes,[^270^, ^271^, ^272^] textile dyes^[273]^ and plasticizers.[^245^, ^274^] Recently, Beckham and co‐workers reported an excellent example of waste PET upcycling.^[261]^ More specifically, reclaimed PET was upcycled into higher‐value reinforced plastics (FRPs), namely an unsaturated polyester and vinyl ester. The upcycled FRPs have a market price of $2.60/lb relative to $0.51 and $0.31/lb for clear and green‐coloured PET flakes, respectively. Additionally, the upcycled FRPs have the potential to realise a 57 % total supply chain energy saving and a reduction in GHG emissions by 40 % over standard petroleum‐derived FRPs. It is therefore clear PET glycolysis represents a tangible route to accessing a diverse range of value‐added products. Moreover, catalysis will undoubtedly underpin the commercial viability of such processes in the future. Whilst we have treated the assessment of organo‐ and metal‐based catalysts in isolation, this is not to say their future application cannot be complementary. Indeed, Dove and co‐workers recently exploited cooperativity between Lewis acid (metal salts) and organic bases for the enhanced glycolysis of PET.^[275]^ Whilst we have aimed to detail major developments in catalytic systems thus far, we acknowledge certain omissions may be of potential use to the scientific community. As such, we direct the interested reader to a thorough Review by Kosloski‐Oh et al.^[276]^ that includes catalytic examples of those omitted, for example polyoxometalates and MOFs. A detailed account of supercritical and microwave‐assisted methods for PET glycolysis is provided by George and Kurian.^[44]^ A summary of catalysts reported for PET glycolysis is provided in Table 3 in addition to an overview of the chemical recycling methods discussed (Figure 12).

**Table 3:** Summary of selected metal‐based and organocatalysts reported for PLA glycolysis.

Cataylst	EG/ester unit (*w*/*w*)	Cat. [wt %]	*T* [°C]	*t* [h]	PET conv. [%]	*Y*_BHET_^[a]^ [%]	Ref.
Organocatalysts							
Urea	4 : 1	10	180	3	100	80	[230]
TBD	5.2 : 1	0.7	190	3.5	100	78	[231]
DMAP	5.2 : 1	16	190	1.67	100	94^[b]^	[235]
DBU	5.2 : 1	13	190	0.11	100	99^[b]^	[235]
DBN	5.2 : 1	15	190	0.12	100	99^[b]^	[235]
TBD/MSA (1 : 1)	20 : 1^[c]^	0.5 : 1^[d]^	180	2	100	91	[237]
ILs							
[Bmim][Cl]	4 : 1	20	180	8	45	–	[238,239]
[Bmim][Br]	4 : 1	20	180	8	99	–	[238,239]
[Bmim][OH]	10 : 1	5	190	2	100	71	[240]
[Bmim]_2_[CoCl_4_]	11.7 : 1	16.7	175	1.5	100	81	[241]
[Ch]_3_[PO_4_]	4 : 1	20	180	4	100	61	[243]
DESs							
4(urea) ⋅ (ZnCl_2_)	4 : 1	5	170	0.5	100	83	[244]
Metal‐based							
ZnCl_2_	10 : 1^[c]^	0.5	197	8	–	73	[247]
NaHCO_3_	6 : 1^[c]^	5	–^[e]^	0.58	–	65	[248]
Zn(OAc)_2_	7.6 : 1^[c]^	1 : 100^[d]^	196	1	–	≈70	[249]
Na_2_CO_3_	7.6 : 1^[c]^	1 : 100^[d]^	196	1	–	≈70	[249]
SO_4_^2−^/ZnO‐TiO_2_‐200–300 °C	5.6 : 1	0.3	180	3	100	72	[250]
β‐zeolite	6 : 1	1	196	8	100	66	[251]
γ‐zeolite	6 : 1	1	196	8	100	65	[251]
Ti(IV)‐phosphate catalyst	2.8 : 1^[c]^	0.3	190–200	2.5	100	98^[b]^	[258]
Ti(OCH_2_CH_2_O)_3_Na_2_	12 : 1^[c]^	1 : 100^[d]^	190	3	–	85	[262]
[Co(dcype)]Cl_2_	11.1 : 1	1.5 : 100^[d]^	190	3	–	75	[263]
[Ni(COD)_2_]/dppe (1 : 2)	11.1 : 1	1 : 100^[d]^	190	3	100	67	[263]
Nanocatalysts							
γ*‐*Fe_2_O_3_	3.3 : 1	0.05	300^[f]^	1	–	>90	[252]
Mn_3_O_4_/SNPs	11 : 1^[c]^	1	300^[f]^	1.33	–	>90	[253,254]
ZnMn_2_O_4_	11.5 : 1^[c]^	1	260^[g]^	1	–	92	[255]
Fe_3_O_4_‐boosted MWCNT	10 : 1	5	190	2	100	100	[256]

[a] *Y*_BHET_ refers to isolated yield of BHET unless otherwise stated. [b] Yield reported as a mass fraction of the products as determined by GPC. [c] Molar ratios are listed. [d] Molar ratio of [catalyst]/[PET]. [e] No temperature reported. Microwave irradiation used, power=800 W. [f] Reaction performed at approximately 1.1 MPa. [g] Reaction performed at 5 atm.

### 4.7 Reductive depolymerisation

**Figure 13:**
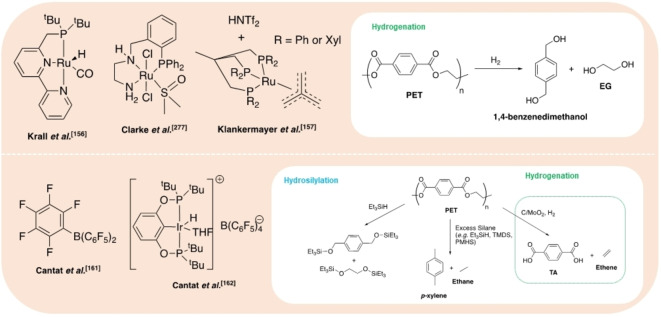
Selected metal‐based and organocatalysts reported for the hydrogenation (top) and hydrosilylation (bottom) of PET.

Metal‐based catalysts exploited in the reductive depolymerisation of PLA (Figure 8) have also been applied to PET. Krall et al.^[156]^ reported the first example of PET hydrogenation mediated by a ruthenium(II)‐PNN pincer complex. Using conditions identical to those discussed for PLA [THF/anisole, 54 bar (H_2_) at 160 °C], bottle‐grade PET was successfully reduced to 1,4‐benzenedimethanol (>99 %) and EG within 24 h. The use of a used water bottle suggests the catalyst is robust to impurities and additives, although the system remains limited by its high energy intensity. Substitution of the pyridine arm for an amine resulted in a loss in catalytic activity, implicating this substituent as an active component in the degradation mechanism. Clarke and co‐workers have previously screened a series of ruthenium(II)‐catalysts bearing tridentate aminophosphine ligands for the hydrogenation of diester model compounds.^[277]^ Product selectivity was found to be dependent on ligand structure, identifying an ethylenediamine variant of a Ru^II^‐sulfoxide complex as the outstanding candidate (Figure 13). Using a solvent mixture of THF and anisole, 73 % conversion to 1,4‐benzenedimethanol was achieved within 48 h under optimal conditions [2 mol % cat, BuOK/cat=20 : 1, 50 bar (H_2_)]. Recently, Klankermayer and co‐workers^[157]^ reported the use of two ruthenium(II)‐complexes bearing tridentate phosphine ligands (triphos and triphos‐xyl) for the hydrogenation of PET in the presence of a co‐catalyst; HNTf_2_ (1 mol %) (Figure 13). Substitution of the phenyl groups with xyl (3,5‐dimethylphenyl) resulted in enhanced PET conversion and selectivity to 1,4‐benzenedimethanol. Promisingly, high conversion (>99 %) and product selectivity (86–99 %) were retained for a variety of commercial PET sources (e. g., bottle, yoghurt pot and sport jersey), demonstrating catalyst robustness. A selective separation method for PET and PLA via catalytic hydrogenation was proposed. Moreover, process scale‐up (>10 g) demonstrated the hydrogenation of PET bottle flake could proceed in the presence of a PP bottle cap and PE label. A drawback of this method are the harsh reaction conditions employed [140 °C, 100 bar (H_2_)] and prolonged reaction times (16 h), although low catalyst loadings are acknowledged (0.2 mol %).

Hydrosilylation methods have also been reported for PET (Figure 13). In 2015, Feghali and Cantat^[161]^ reported a two‐step catalytic process using B(C_6_F_5_)_3_ (2 mol %) for the production of 1,4‐benzenedimethanol, characterised by hydrosilylation followed by hydrolysis. Using Et_3_SiH, green PET flakes were converted into two silyl ethers, namely silylated 1,4‐benzenedimethanol (85 %) and EG (72 %), within 3 h at RT. The formation of such silyl ethers is attractive since they can be used as sources of alkoxide groups in Ullman's coupling reactions to prepare ethers.^[278]^ These disilylethers were subsequently hydrolysed to 1,4‐benzenedimethanol and EG using 2.1 equiv. of TBAF ⋅ 3H_2_O. 1,4‐Benzenedimethanol is a valuable building block for the production of pesticides, perfumes and dyes and is directly accessible via the aforementioned hydrogenation routes. In the presence of excess silane and at high catalyst loadings, PET could be converted into *p‐*xylene and ethane in up to 49 % yield under prolonged stirring (16 h). Enhanced *p‐*xylene yields of 82 and 75 % were realised upon substituting Et_3_SiH for TMDS and PMHS, respectively. High catalyst tolerance was demonstrated in the presence of mixed waste feeds, which included PLA and PVC. Moreover, the reaction conditions employed are significantly milder relative to previously reported systems, thus lending itself to industry. However, B(C_6_F_5_)_3_ is limited by a high cost comparable to precious rare earth metals. Subsequent work in this group has utilised Brookhart's iridium(III) catalyst for PET hydrosilylation.^[162]^ Lower catalyst loadings (1 mol %) are noted relative to B(C_6_F_5_)_3_, albeit at the expense of elevated temperatures (70 °C) and prolonged reaction times (72 h). Moreover, hydrosilylation products of PET were isolated in lower yields (48–63 %). Catalyst versatility was demonstrated by application to the hydrosilylation of polycarbonates (PPC and BPA‐PC), which typically proceeded more rapidly relative to polyesters at lower catalyst loadings. The use of undesirable toxic halogenated solvents (CH_2_Cl_2_ and chlorobenzene) is noted in both systems, respectively.[^161^, ^162^] The environmental impact of toxic solvent waste is of particular concern upon upscaling. Recently, Nunes et al.^[163]^ reported a cheap and air‐stable dioxomolybdenum complex, MoO_2_Cl_2_(H_2_O)_2_. Employing PhSiH_3_ as the reducing agent, *p‐*xylene could be obtained in 65 % yield after 4 days under notably harsher reaction conditions than those previously reported (chlorobenzene, 160 °C). No evidence of 1,4‐benzenedimethanol as an intermediate was observed and prolonged reaction (7 days) resulted in complete disappearance of the NMR signal pertaining to EG, suggesting reduction to ethane. High catalyst and silane loadings of 5 and 6 mol %, respectively, were used, although potential economic benefits offset low catalyst activity. Indeed, such work provides scope for future optimisation. Catalyst tolerance was demonstrated using multiple sources of PET (e. g., bottle, sport jersey and pillow filling), maintaining reasonable *p‐*xylene yields (62–65 %). In 2020, Marks and co‐workers reported a carbon‐supported single‐site molybdenum‐dioxo catalyst (C/MoO_2_) for the reduction of PET to TA and EG.^[279]^ Using a PET+PP system to model a bottle, 87 % yields of TA, ethylene and trace acetaldehyde (<5 %) were observed within 24 h at 260 °C (1 atm H_2_, Ester/Mo=40 : 1). Catalyst stability and recyclability was successfully demonstrated, averaging 90 % TA yield over 4 consecutive runs (24 h, 260 °C, 1 atm H_2_, Ester/Mo=40 : 1). A summary of the systems discussed is provided in Table 4.

**Table 4:** Summary of selected metal‐based and organocatalysts reported for the hydrogenation and hydrosilylation of PET.

Cataylst	Cat. [mol %]	Solvent	*T* [°C]	H_2_ [bar]	*t* [h]	PET conv. [%]	Product(s)	Yield^[a]^ [%]	Ref.
Hydrogenation									
ruthenium(II)‐PNN pincer	2^[b]^	anisole/THF	160	54.4	24	100	1,4‐benzenedimethanol; EG	>99	[156]
Ru^II^‐triphos complex (R=xyl)	0.2^[c]^	1,4‐dioxane	140	100	16	100	1,4‐benzenedimethanol; EG	>99	[157]
Ru^II^‐sulfoxide complex	2^[d]^	anisole/THF	110	50	48	–	1,4‐benzenedimethanol; EG	73	[277]
C/MoO_2_	2.5	–	260	1.0	24	–	TA; EG; trace acetaldehydes	87; 87; <5	[279]
Hydrosilylation				Silane (equiv.)					
B(C_6_F_5_)_3_	2	CH_2_Cl_2_	25	Et_3_SiH (4.3)	3	–	Si‐1,4benzenedimethanol; Si‐EG	85; 72^[e]^	[161]
	5	CH_2_Cl_2_	25	TMDS (6.0)	16	–	*p‐*xylene; ethane	82; –^[f]^	[161]
	7.5	CH_2_Cl_2_	25	PMHS (11.0)	16	–	*p‐*xylene; ethane	75; –^[f]^	[161]
Brookhart's iridium(III) catalyst	1	chlorobenzene	70	Et_3_SiH (6.0)	72	–	Si‐1,4benzenedimethanol; Si‐EG	63; 48^[e]^	[162]
MoO_2_Cl_2_(H_2_O)_2_	5	chlorobenzene	160	PhSiH_3_ (6.0)	96	–	*p‐*xylene; EG	65	[163]

[a] Product yields refer to ^1^H NMR analysis unless otherwise stated. [b] KO*t*Bu employed as a co‐catalyst in a loading ratio of 50 : 1 : 2 {[ester repeat unit]/[catalyst precursor]/[KO*t*Bu]}. [c] HNTf_2_ employed as a co‐catalyst in a loading ratio of 500 : 1 : 5 {[ester repeat unit]/[catalyst precursor]/[HNTf_2_]}. [d] KO*t*Bu employed as a co‐catalyst in a loading ratio of 50 : 1 : 20 {[ester repeat unit]/[catalyst precursor]/[KO*t*Bu]}. [e] Isolated yield. [f] *p‐*Xylene yield obtained by GC‐MS analysis. Ethane yield not determinable by ^1^H NMR spectroscopy owing to insolubility of PET in CH_2_Cl_2_.

### 4.8 Other products

The catalytic pyrolysis of PET remains underexplored as solvolysis methods generally offer superior product selectivity. Typically, high temperature regimes (400–700 °C) are used, notably higher relative to PLA. Moreover, the resulting degradation feeds are often complex mixtures of solids, liquids and gases that require costly and extensive separations. The impregnation of simple metal salts (e. g., CuCl_2_) have been shown to dramatically increase the extent of PET cracking.^[280]^ The use of calcium oxide and calcium hydroxide, among other metal salts, produces benzene‐rich oils with a significantly higher benzene content relative to thermal pyrolysis.[^281^, ^282^, ^283^, ^284^] Indeed, product distribution has been shown to be highly dependent on the metal oxide catalyst employed.^[282]^ For example, a mixture of Ca(OH)_2_ and NiO favoured the formation of synthesis gas (CO+H_2_), which is an important building block used in numerous industrial processes, perhaps most notably the Fischer–Tropsch process for hydrocarbon production.^[285]^ Conversely, a considerable reduction in gaseous products was observed using TiO_2_. A notable drawback of this method is the production of sublimate materials such as TA and benzoic acid, which can result in pipe blockages leading to plant downtime. To this end, Masuda et al.^[284]^ demonstrated FeOOH as a cheap catalyst that yields no sublimate material, highlighting the importance of catalyst design in circumventing by‐product production. Recently, El‐Sayed and Yuan^[286]^ provided an excellent account of using waste plastic, including PET, as a source of organic linker in the production of MOFs. Such materials have broad applicability ranging from gas storage and separation through to catalysis and sensing.

## 5 Emerging Materials

In the preceding sections we have highlighted chemical recycling strategies for two commercial polyesters, namely PLA and PET. For such materials, the development of future waste management strategies relies on retrospective action to combat plastic pollution. However, as the plastic industry transitions to a low‐carbon and circular future, it is imperative recyclability is embedded at the design phase. In this final section, we aim to highlight key contributions and promising developments in this area.

### 5.1 Covalent adaptable networks

Thermoset materials are widely used in demanding engineering applications owing to their high mechanical strength and elasticity. Such favourable properties are derived from cross‐linking via permanent covalent networks, which render the material unsuitable for physical or solution processing. Consequently, material down‐cycling via mechanical processing is often the optimal outcome.^[22]^ A promising solution to PCW thermosets is the use of covalent adaptable networks (CANs). Such dynamic covalent networks are reversible in nature and can be controllably biased in accordance to a stimuli response such as light, heat or pH.[^287^, ^288^, ^289^] Indeed, the dynamic bonds provide exchangeable anchor points within the network to facilitate material remoulding and repair with the potential for self‐healing and retention of structural integrity.[^22^, ^290^] Numerous CAN systems have been reported to date, which include carbonate,^[291]^ imine,[^292^, ^293^] urea,^[294]^ ester^[295]^ and thioester motifs.^[296]^ Despite the vast array of CAN materials reported, complete depolymerisation to monomer(s) remains challenging. Additionally, the presence of such dynamic networks complicates further transformations in the overall recycling process. Nonetheless, monomer recovery has been reported for hemiaminal^[297]^ and boroxine^[298]^ systems among other motifs.^[22]^

**Scheme 4:**
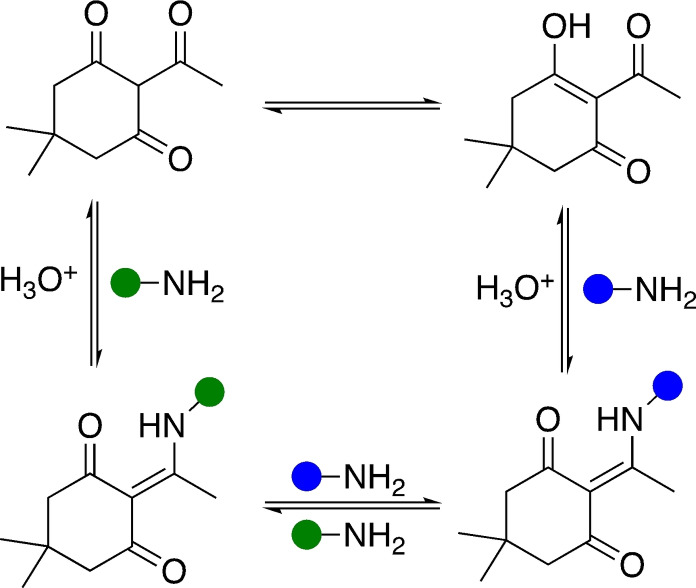
Dynamic diketoenamine bonds for the production of a CAN material.^[299]^

Helms and co‐workers recently reported the closed‐loop recycling of plastics enabled by dynamic covalent diketoenamine bonds (Scheme 4).^[299]^ Promisingly, the starting monomers could be efficiently recaptured and isolated from additives (e. g., dyes, inorganic fillers, flame retardants) and fibres present in the poly(diketoenamine)s (PDKs) under strongly acidic conditions in water. The potential to decouple monomers from material additives will undoubtedly assist market penetration in the future. Current work in the field remains focused on improving the overall sustainability of chemically recyclable thermosets and we direct the interested reader to a recent Review by Worch and Dove.^[22]^

### 5.2 Self‐immolative polymers

**Scheme 5:**
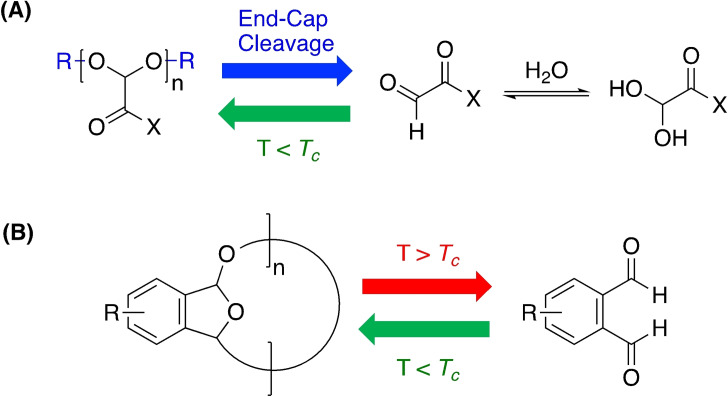
Example SIPs: (A) Polygloxyls and (B) PPA.^[22]^

Self‐immolative polymers (SIPs) have attracted considerable interest in recent years owing to their ability to “trigger” complete depolymerisation for on‐demand material disposal applications.^[300]^ SIP degradation is typically irreversible in nature, akin to biodegradable polymers, although chemical recyclability is possible when monomeric units are recovered. Traditionally, reversible SIPs exhibit a low *T*_c_, observing polymer stability below this temperature. Cleavable end‐capping units have been shown to provide sufficient chain stability above *T*_c_ for SIPs with extremely low ceiling temperatures (*T*_c_<20 °C), enabling practical applications.[^22^, ^300^] Examples of SIPs include polyglyoxylates^[301]^ and polyphthalaldehydes (PPA) as shown in Scheme 5.^[302]^ We envision such materials will play an important role in the future economy owing to their ability to exhibit well‐defined complete depolymerisation. This is a major limitation of current biodegradable polymers, which exhibit differing degradation profiles depending on a variety of external environmental factors, including temperature and humidity.

Since non‐composite SIPs typically exhibit poor mechanical properties, recent work in the field has focused on material property enhancement. A recent example includes the development of a thermally robust PPA with potential applications as a thermoplastic material.^[303]^ Zimmerman and co‐workers recently reported a trigger‐responsive self‐amplifying degradable polymer based on a 3‐iodopropyl acetal moiety.^[304]^ Acid catalysed hydrolysis promotes chain cleavage and liberation of the triggering species in stoichiometric quantities, resulting in accelerated degradation. Indeed, mechanically initiated chain scission via sonication has also been reported.[^305^, ^306^]

Beyond this, polyphosphoesters (PPEs), such as poly(methyl ethylene phosphate) (PMEP) and poly(ethyl ethylene phosphate) (PEEP), have also been reported as SIPs.^[307]^ Such poly(alkyl ethylene phosphate)s were shown to undergo backbiting hydrolysis under basic conditions, liberating alkyl (2‐hydroxyethyl) hydrogen phosphate as the primary degradation product. PPEs are used in a diverse range of applications from flame retardants and tissue engineering through to drug and gene delivery systems.^[308]^

### 5.3 Polyolefin mimics

The underlying thermodynamics of highly exergonic polymerisations (e. g., olefins) ensures reversing such transformations will remain challenging. A possible solution to this is the development of polyolefin mimics. Such materials retain many of the revered properties of polyolefins but are less environmentally persistent owing to the presence of cleavable linkages. Examples include polyphosphonates^[309]^ and polyesters^[310]^ among others.^[311]^ Recently, Wurm and co‐workers reported long‐chain polyorthoesters^[312]^ and polypyrophosphates^[313]^ as degradable alternatives to PE. Post polymerisation hydrogenation of the polyorthoesters yielded hard, solid materials with thermal properties similar to PE. Hydrogenated and non‐hydrogenated co‐polymers were found to hydrolyse slowly when exposed to atmospheric moisture, the rate of which was dependent on the orthoester substituent in solution. Conversely, the polypyrophosphates were found to hydrolyse rapidly under neutral, basic and acidic conditions. These materials have potential applications in the biomedical field or for advanced packaging. Whilst promising, it is important to note such materials do not address the loss of embedded material value to the natural environment or eutrophication resulting from nutrient saturation.

### 5.4 Monomer diversification

**Scheme 6:**
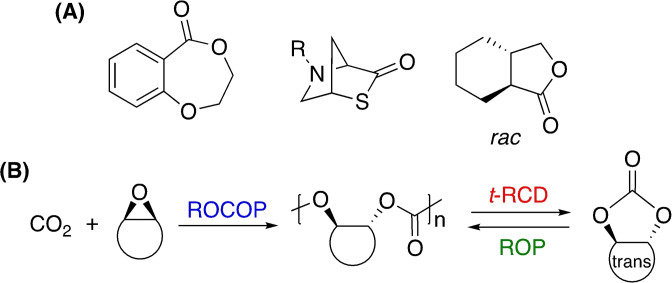
(A) Selected lactone monomers that can undergo ROP and CRM.^[17]^ (B) Polycarbonate synthesis via ROCOP followed by RCD to afford *trans*‐[4.3.0] carbonates.

In pursuit of a plastics economy decoupled from fossil fuels, monomer sourcing considerations are becoming increasingly important. Additionally, it is imperative the industries pursuit of sustainability and circularity informs the selection of appropriate alternative feedstocks. Coates and Getzler^[17]^ recently defined the most attractive ROP monomers for chemical recycling to monomer (CRM) as large rings (7–11‐membered) or five‐ and six‐membered rings that possess multiple non‐sp^3^‐hybridised atoms or ring fusions (Scheme 6a). Such structural features increase Δ*H*_p_, affording polymers that lend themselves to chemical recycling. We identify the economic derivatisation of such monomers from biomass as a key challenge in the future. Indeed, it is conceivable to access such monomers via the CRM of polymer derived from a different initial feedstock. For example, *trans*‐[4.3.0] carbonates could be sourced from the ring‐closing depolymerisation (RCD) of polycarbonates produced via the ring‐opening copolymerisation (ROCOP) of CO_2_ and epoxide (Scheme 6b).^[17]^ Whilst high monomer cost relative to cheap petrochemical feedstocks has limited bio‐based plastics thus far, cost is expected to decrease with scale‐up. Indeed, the valorisation of polymer waste through CRM or upcycling may assist in overcoming a high initial monomer cost.

### 5.5 Cost and performance competitiveness

**Scheme 7:**
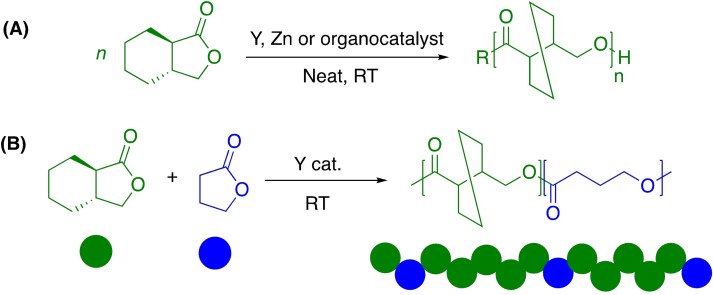
Chemically recyclable bio‐based homopolymer (A) and copolymer (B) based on a fused ring *γ*‐butyrolactone monomer.^[316]^

**Scheme 8:**
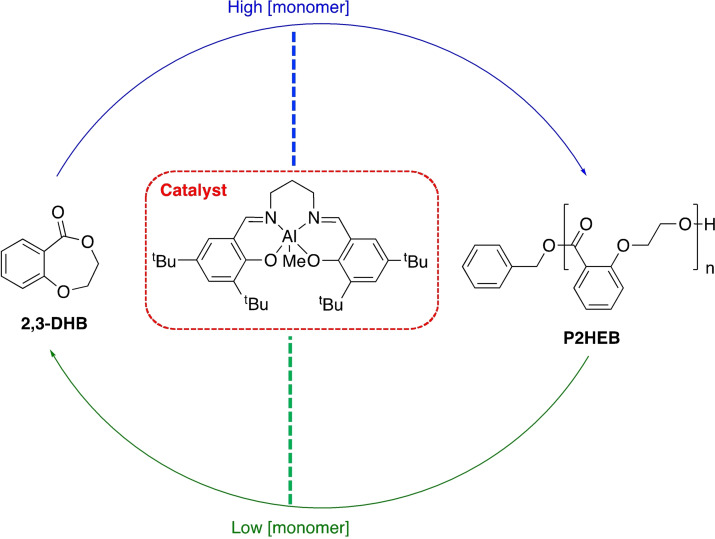
A fully recyclable aromatic bio‐based polyester based on the ROP of 2,3‐dihydro‐5*H*‐1,4‐benzodioxepin‐5‐one (2,3‐DHB) mediated by an Al^III^‐salen complex.^[317]^

Whilst the plastic economies transition relies on the development of new materials, it is imperative they remain cost‐ and performance‐competitive with existing synthetic plastics. Desirable material properties of emerging plastics include: a low glass transition temperature (*T*_g_), high melting temperature (*T*_m_), good ductility and high tensile strength.^[22]^ Whilst significant effort is devoted to advancing this research front, their inherent recyclability must not be overlooked to avoid potential pitfalls. Significant advancements with regards to bio‐based polyesters have been made in recent years. Chen and co‐workers reported a polyester based on *γ*‐butyrolactone with *trans*‐ring fusion at the *α‐* and *β*‐positions. Such *trans*‐ring fusion renders the monomer polymerizable at room temperature under solvent‐free conditions in the presence of a transition‐metal (e. g., yttrium or zinc)^[314]^ or organocatalyst^[315]^ (Scheme 7a). Promisingly, the resulting polymer could be repeatedly recycled by means of thermolysis or chemolysis, recovering monomer in quantitative yield.^[314]^ Subsequently, this monomer has been copolymerised with a cyclic lactone (Scheme 7b) to afford a chemically recyclable copolymer with barrier and mechanical properties competitive with existing commodity plastics such as PE and PET.^[316]^ Sustainable and recyclable biopolyesters reported thus far typically employ an aliphatic backbone and examples of novel aromatic polyesters remain rare despite a clear market need, exemplified by PET. Shaver and co‐workers[^317^, ^318^] have reported a novel aromatic polyester with complete chemical recyclability back to monomer mediated by an Al^III^‐salen catalyst (Scheme 8). Such examples offer the prospect of a future plastics economy portfolio of robust and chemically recyclable plastics, surmounting the expectations of current first generation biopolyesters derived from cyclic lactones such as lactide.[^17^, ^22^]

## 6 Conclusions and Outlook

Despite mounting environmental concerns, plastics will continue to play a dominant role in human development for the foreseeable future. It is therefore of critical importance we adopt proactive action to deliver disruptive and transformative change within a meaningful timeframe. Central to this notion is the decoupling of plastics from depleting fossil reserves and a shift towards a circular economic model, one concerned with material recapture and reuse. This will require the development of alternative waste management strategies, for which there is a clear industry appetite. Recycling represents a promising enabler to this transition. Mechanical recycling remains the industry standard but is limited by eventual material downcycling, which creates uncertainty surrounding retention of material‐value in the long‐term. A possible solution to this is chemical recycling, which encompasses depolymerisation to monomer and degradation to value‐added products. Examples from this Review include the derivatisation of alkyl lactates (e. g., green solvent) and terephthalamides (e. g., building blocks for high performance materials) from poly(lactic acid) (PLA) and poly(ethylene terephthalate) (PET) waste respectively. The potential to realise enhanced economic performance will undoubtedly play a crucial role in overcoming inevitable barriers to adoption within industry. Additionally, catalysis will likely underpin the commercial viability of such processes and we have highlighted recent developments concerning PLA and PET. Despite recent progress, it is clear current methods remain limited by a number of factors including the use of expensive and/or highly corrosive reagents, harsh operating conditions or prolonged reaction times. Catalyst recovery often remains overlooked and the impact of mixed plastic waste on catalyst activity and product separation remains poorly understood. Such challenges provide scope for future process optimisation with metal‐based catalysis a possible solution, although literature examples remain limited. In accordance with criteria previously described by Worch and Dove,^[22]^ we propose the following targets to encourage the development of industrially viable and sustainable chemical recycling strategies using metal‐based systems:

1. exploit the use of cheap and earth‐abundant metals in combination with scalable ligands
2. simple catalyst recovery and reuse, maintaining performance between cycles both in batch or flow
3. robust catalysts tolerant to common plastic waste stream contaminants including additives and debris
4. high process efficiency under mild conditions (≥90 %, <100 °C, ≤1 h)
5. maintain high product selectivity (≥90 %, <10 % per plastic) and process efficiency in the presence of mixed plastics.

It is clear a “one‐solution‐fits‐all” approach is unrealistic, and we expect such criteria to direct the development of a diverse array of chemical recycling strategies. Additionally, future catalyst design should pursue the incorporation of Lewis acidic and H‐bonding motifs, factors known to promote enhanced degradation activity. Whilst we anticipate mixed plastic waste to remain a major challenge, we expect the emergence of switchable catalysis (e. g., photo‐ and electrochemically induced) to offer new solutions to such problems. It is important to recognise a future circular model will be imperfect and thus susceptible to leakage. Embedding polymer recyclability and biodegradability at the design phase will assist in circumventing such challenges. Moving forward, it is imperative such materials remain cost and performance competitive with existing synthetic plastics. Moreover, we do not expect future innovation to be limited to the plastic materials themselves and anticipate developments in reaction engineering, and so forth, to assist in the transition.^[319]^ As the field garners increasing momentum, industry can expect significant advancements within the next 10 years. It is therefore prudent lessons learned from PLA and PET be applied to existing and emerging materials, for example poly(ethylene furanoate) (PEF).^[11]^ Whilst many catalytic chemical recycling processes remain immature relative to established thermal pyrolytic methods, we remain optimistic of its potential to modernise the plastics economy. Indeed, British Petroleum (BP) recently announced the development of its BP Infinia recycling technology for unrecyclable PET, highlighting the field's industrial relevance.^[320]^ Finally, it is imperative policy and legislation endeavour to deliver a platform that provides continuity between all invested stakeholders. This will remove barriers that currently confound the plastic waste crisis and accelerate the uptake and implementation of such technology.

## Conflict of interest

The authors declare no conflict of interest.

## Biographical Information

*Jack Payne is a Ph.D. student at the Centre for Sustainable and Circular Technologies (CSCT). Jack holds master's degrees (hons) in Chemistry (First Class) and Sustainable Chemical Technologies (Distinction) from the University of Bath. He has previous industrial experience working for Shell on the development of novel diesel performance additives. His current research spans both chemistry and engineering, focusing on the application of metal‐based catalysis for renewable polymer production and chemical recycling of plastics*.

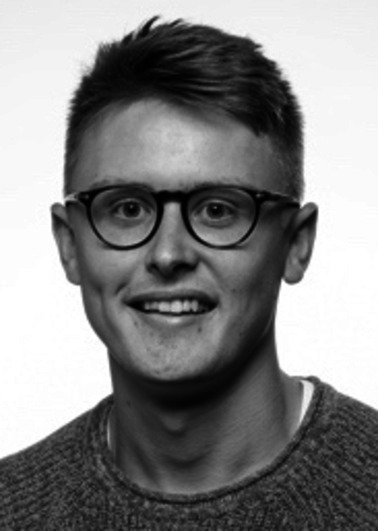

## Biographical Information

*Matthew Jones is a Professor of Inorganic Chemistry at the University of Bath. He obtained his Ph.D. in 2003 under the supervision of Professors Brian Johnson and Melinda Duer at Cambridge University. After a brief sojourn in industry he moved back to academia in 2004 as a PDRA in the group of Professor Matthew Davidson at Bath, where he remained being promoted from research fellow (2007), lecturer (2012), senior lecturer (2012), reader (2017) and finally to Professor (2019). He has graduated 13 Ph.D. students and his current work focusses on novel catalysts for polymer production and degradation*.